# CC-115, a dual inhibitor of mTOR kinase and DNA-PK, blocks DNA damage repair pathways and selectively inhibits ATM-deficient cell growth *in vitro*

**DOI:** 10.18632/oncotarget.20342

**Published:** 2017-08-18

**Authors:** Toshiya Tsuji, Lisa M. Sapinoso, Tam Tran, Bonny Gaffney, Lilly Wong, Sabita Sankar, Heather K. Raymon, Deborah S. Mortensen, Shuichan Xu

**Affiliations:** ^1^ Oncology Research, Celgene Corporation, San Diego, CA 92121, USA; ^2^ Pharmacology, Celgene Corporation, San Diego, CA 92121, USA; ^3^ Medicinal Chemistry, Celgene Corporation, San Diego, CA 92121, USA

**Keywords:** mTOR kinase, DNA-PK, DNA damage repair, ATM

## Abstract

CC-115, a selective dual inhibitor of the mammalian target of rapamycin (mTOR) kinase and DNA-dependent protein kinase (DNA-PK), is undergoing Phase 1 clinical studies. Here we report the characterization of DNA-PK inhibitory activity of CC-115 in cancer cell lines. CC-115 inhibits auto-phosphorylation of the catalytic subunit of DNA-PK (DNA-PKcs) at the S2056 site (pDNA-PK S2056), leading to blockade of DNA-PK-mediated non-homologous end joining (NHEJ). CC-115 also indirectly reduces the phosphorylation of ataxia-telangiectasia mutated kinase (ATM) at S1981 and its substrates as well as homologous recombination (HR). The mTOR kinase and DNA-PK inhibitory activity of CC-115 leads to not only potent anti-tumor activity against a large panel of hematopoietic and solid cancer cell lines but also strong induction of apoptosis in a subset of cancer lines. Mechanistically, CC-115 prevents NHEJ by inhibiting the dissociation of DNA-PKcs, X-ray repair cross-complementing protein 4 (XRCC4), and DNA ligase IV from DNA ends. CC-115 inhibits colony formation of ATM-deficient cells more potently than ATM-proficient cells, indicating that inhibition of DNA-PK is synthetically lethal with the loss of functional ATM. In conclusion, CC-115 inhibits both mTOR signaling and NHEJ and HR by direct inhibition of DNA-PK. The mechanistic data not only provide selection of potential pharmacodynamic (PD) markers but also support CC-115 clinical development in patients with ATM-deficient tumors.

## INTRODUCTION

The mammalian target of rapamycin (mTOR), a serine/threonine kinase, is a member of the phosphoinositide 3-kinase related kinase (PIKK) family that includes ataxia-telangiectasia mutated (ATM), ataxia-and Rad3-related (ATR), the catalytic subunit of DNA-dependent protein kinase (DNA-PKcs), suppressor of morphogenesis in genitalia (SMG1), and transformation / transcription domain-associated protein (TRRAP). mTOR functions in two distinct multiprotein complexes: mTORC1 which consists of raptor, LST8, PRAS40, and Deptor and mTORC2 which includes rictor, LST8, SIN1, Deptor, and Protor [1, 2]. mTORC1 integrates signals from growth factor receptors with cellular nutritional status and controls the level of mRNA translation by modulating the activity of key translational components including 4E-BP1 and p70S6 kinase. mTORC2 is thought to modulate growth factor signaling by phosphorylating kinases such as AKT, which leads to its activation. Active AKT promotes cell survival in many ways, including suppressing apoptosis, promoting glucose uptake, and modifying cellular metabolism [3]. The PI3K–AKT pathway is inappropriately activated in many cancers and is vital to the growth and survival of cancer cells [4]. Therefore, mTOR inhibitors are being developed as potential cancer therapeutic agents. Rapamycin analogs (rapalogs) temsirolimus and everolimus have been approved by FDA for treatment of different indications including renal cell carcinoma (RCC), pancreatic neuroendocrine tumors, certain TSC-related tumors and, as part of a combination therapy, hormone-positive, HER2-negative breast cancer [5]. Rapalogs do not directly inhibit mTOR kinase activity and, consequentially, only inhibit some function of the mTORC1 complex such as phosphorylation of p70S6 kinase, but have limited inhibitory effect on 4E-BP1, a critical substrate of mTORC1 [5]. In addition, rapalogs stimulate the upstream kinase AKT by inhibiting the negative feedback loop between p70S6 kinase and Insulin Receptor Substrate 1 (IRS1), which may limit their clinical utility [6, 7]. The second generation ATP-competitive kinase-targeting mTOR inhibitors (OSI-027 [8], INK-128 [9], and CC-223 [10]) inhibit both mTORC1 and mTORC2 complexes and are currently being tested in clinical studies for cancer treatment. These mTOR kinase inhibitors have broad and potent anti-proliferation activities in preclinical models but have shown only modest clinical activities in early trials [11, 12]. One potential reason is inhibition of mTORC1 and mTORC2 triggers several feedback loops which may promote tumor growth [13, 14].

DNA-PK, composed of DNA-PKcs (also a member of the PIKK family) and two regulatory subunits Ku70 and Ku80, is essential for repair of double-strand DNA (dsDNA) mediated by non-homologous end-joining (NHEJ). In mammalian cells, dsDNA breaks (DSBs) are mainly repaired through two distinct pathways: homologous recombination (HR) and NHEJ. HR is a complex, error-free pathway that requires alignment of the broken DNA ends with a homologous region on the sister chromatid [15]. NHEJ, which repairs the majority of DSBs, is the process of rejoining broken DNA ends without reference to a second template [15, 16]. The core NHEJ machinery consists of the Ku70/80 heterodimer, DNA-PKcs, DNA ligase IV, XRCC4, and the endonuclease Artemis. The broken ends of dsDNA are first recognized and bound by Ku70/80 which recruits DNA-PKcs. DNA-PKcs acts as a scaffold protein to aid the localization of other DNA repair proteins including XRCC4, XLF, DNA ligase IV, and the endonuclease artemis to the site of DNA. Artemis processes the DNA ends, and the final ligation of the juxtaposed ends is accomplished by the XRCC4/XLF/DNA ligase IV complex [17, 18]. Once DNA-PKcs has formed a complex with Ku70/80 at DNA, DNA-PK becomes active and phosphorylates itself at S2056 and other sites, as well as a wide range of DNA damage/checkpoint proteins including Artemis, XRCC4 and DNA ligase 4 [17].

Cancer cells display increased DSBs due to replication stress driven by excess proliferation [19–21] and therefore rely on DNA damage repair pathways for survival. Loss of DNA-PK or inhibition of DNA-PK activity leads to increased sensitivity of cancer cells to DNA damaging agents such as radiation and chemotherapies [22–24]. In addition, ATM-deficient cancer cells show strong addiction to DNA-PK [25]. Therefore, DNA-PK is an attractive target for cancer therapy when used in combination with existing chemo-/radio-therapy to potentially achieve synergistic effects. CC-115 is a dual inhibitor of both mTOR kinase and DNA-PK that is currently undergoing Phase I studies [26, 27, 52]. Here, we demonstrate that CC-115 is a selective mTOR kinase/DNA-PK dual inhibitor with excellent kinase selectivity, even over closely related ATR, ATM and PI3K-alpha and with potent inhibitory activity across many cancer cell lines. We further describe the molecular mechanism of action of CC-115 in inhibition of DNA repair pathways, which supports the identification of pharmacodynamic (PD) markers. Finally, we describe the identification of patients with ATM-deficient tumors for CC-115 clinical development.

## RESULTS

### CC-115 inhibits mTOR kinase and DNA-PK activity in cancer cells

**Figure 1:**
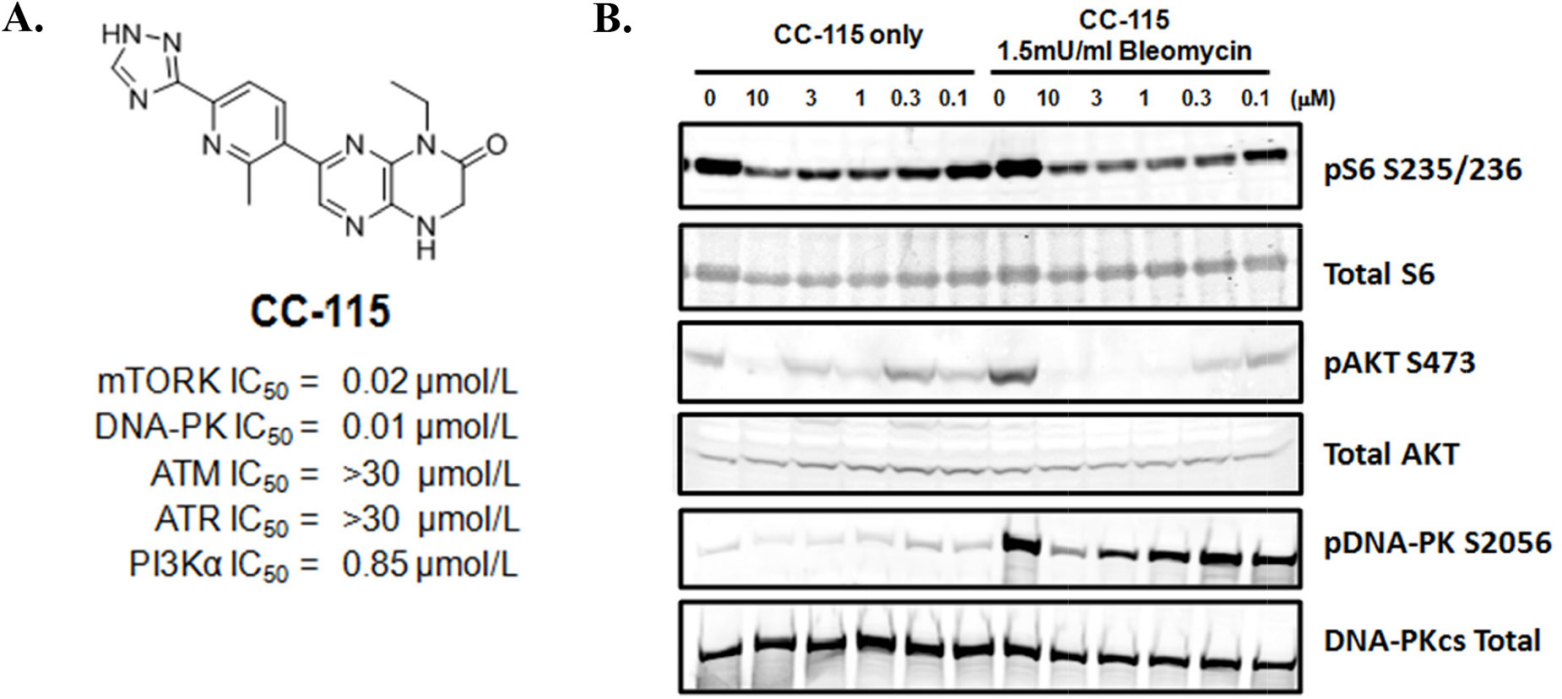
CC-115 inhibits mTOR and DNA-PK in NCI-H441 cells **(A)** Structure and PIKK kinase potency for CC-115. **(B)** Western blot analysis of NCI-H441 lung cancer cells treated with CC-115 at different concentrations (0-10μM) in absence or presence of 1.5mU/mL bleomycin for 2 hrs.

CC-115 is a dual inhibitor of mTOR kinase and DNA-PK with enzyme IC_50_ values of 21 and 13 nM, respectively (Figure 1A and Supplementary Table 1). CC-115 is selective for mTOR kinase and DNA-PK, showing ∼40-fold selectivity over the related lipid kinase PI3K-alpha (IC_50_=850 nM) and other PIKK family members tested (ATM and ATR, IC_50_ >30 μM) and further demonstrates general kinome selectivity, inhibiting only 2 of 250 protein kinases in an expanded selectivity panel [26]. In cells, formation of pS6 S235/236, pAKT S473, and pDNA-PKcs S2056 were used as biomarkers to evaluate the inhibition by CC-115 of mTORC1, mTORC2, and DNA-PK activity, respectively. In NCI-H441 cells, CC-115 potently suppresses mTORC1 biomarker pS6 S235/236 in the absence of DNA damage-inducing agent bleomycin (Figure 1B). Bleomycin treatment does not change pS6 S235/236 (lane 1 vs 7) and the inhibition of pS6 S235/236 by CC-115 was not altered by bleomycin treatment. On the other hand, bleomycin increases the mTORC2 biomarker pAKT S473 in NCI-H441 and CC-115 potently reduces basal and bleomycin-stimulated pAKT S473. The basal pDNA-PK S2056 level is low but inhibition of basal pDNA-PK S2056 by 3 and 10 μM CC-115 can be detected (Figure 1B, lanes 1-6). As expected, the DNA damage-inducing agent bleomycin significantly increases the level of pDNA-PK S2056 and CC-115 inhibits bleomycin-induced pDNA-PKcs S2056 in a concentration-dependent manner (Figure 1B, lanes 7-12). Quantitation of the Western blots from multiple experiments indicates that in the presence of bleomycin CC-115 inhibits pS6 S235/236, pAKT S473, and pDNA-PK S2056 with average IC_50_ values (n=3) of 0.16 ±0.01 μM, 0.136 ± 0.062 μM and 2.6 ± 0.45 μM, respectively. These data indicate that CC-115 inhibits both mTOR kinase and DNA-PK in cells but its cellular activity against DNA-PK is ∼16-fold weaker than its activity against mTOR kinase. Similar results were observed in Hop92 lung cancer cell line (Supplementary Figure 9).

### CC-115 potently inhibits proliferation and induces apoptosis in many cancer cell lines

**Figure 2:**
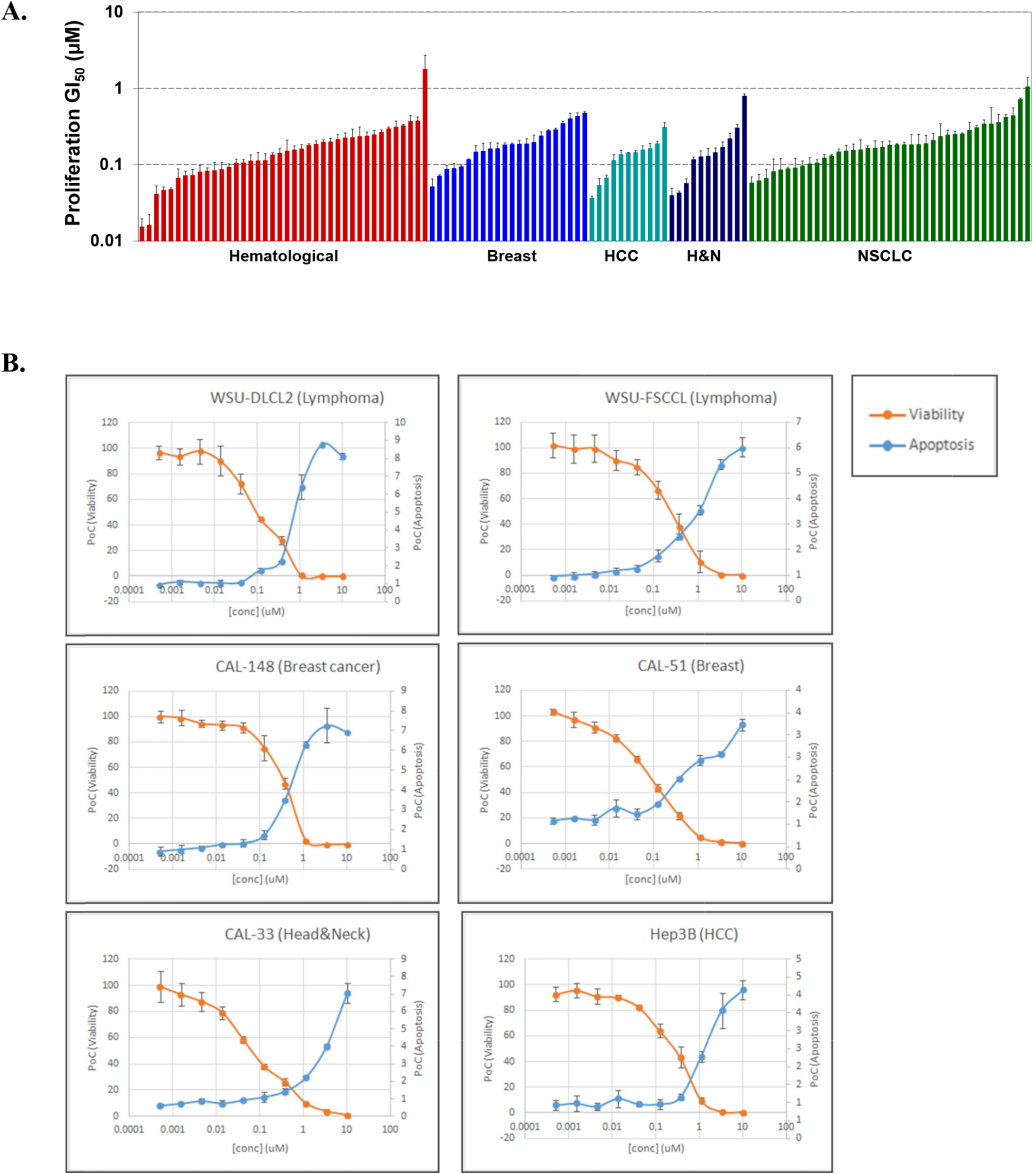
CC-115 potently inhibits the proliferation and induces apoptosis of many cancer cell lines **(A)** Cellular growth inhibition for CC-115 across a panel of 123 cell lines of hematological (red), breast (blue), hepatocellular (teal), head and neck (dark blue) and non small cell lung cancer (green) tumor types. **(B)** Apoptosis induction by CC-115 in both hematological and solid tumor lines.

To evaluate the impact of CC-115 inhibition of mTOR kinase and DNA-PK, CC-115 was tested *in vitro* across a panel of 123 cancer cell lines composed of 40 lymphoma and leukemia, 22 breast cancer, 11 hepatocellular carcinoma, 11 head and neck cancer, and 39 lung cancer cell lines. These cell lines were chosen based on Celgene clinical development strategy for CC-115, the potential roles of mTOR and DNA-PK in these tumor types, and commercial availability of the cell lines. CC-115 has potent growth inhibitory activity against the majority of the cancer cell lines with GI_50_ values ranging from 0.015 μM to 1.77 μM (Figure 2A and Supplementary Table 2). While selective inhibitors of mTOR kinase have been reported to primarily result in cell cycle arrest without significant induction of apoptosis in solid tumor lines [7, 10], in a subset of both hematological and solid tumor cell lines, CC-115 induced strong apoptosis. Representative examples are shown in Figure 2B.

### CC-115 inhibits NHEJ in a reconstituted cell-free system and in intact cells

**Figure 3:**
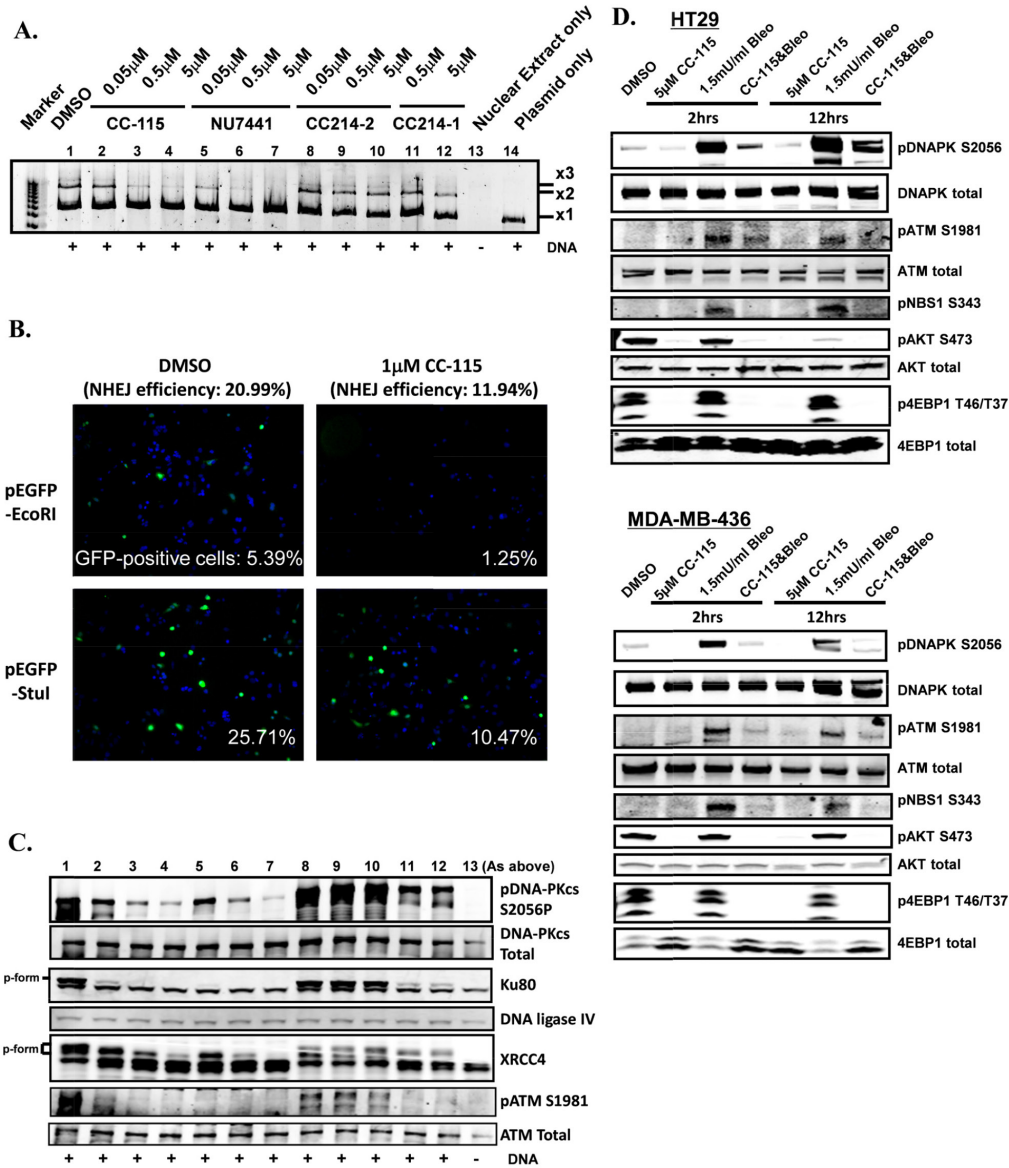
CC-115 inhibits NHEJ activity **(A)** Inhibition of NHEJ in a cell-free assay by CC-115 and NU7441 but not the mTOR kinase selective CC214-2. **(B)** Inhibition of NHEJ in Cal-51 cells, as assessed by a GFP-reporter gene by CC-115 at 1 μM. **(C)** Inhibition of phosphorylation of DNA-PK and its substrates Ku80 and XRCC4, in addition to ATM in the cell-free extracts (from A) as assessed by Western blot. **(D)** Inhibition of mTOR, DNA-PK and ATM biomarkers in HT29 and MDA-MB-436 cell lines. The experiments were independently performed at least 3 times and representative data are presented.

The functional impact of DNA-PK inhibition by CC-115 was determined first using a reconstituted cell-free NHEJ assay. Nuclear proteins were extracted from the MCF7 breast cancer cell line and enrichment of NHEJ-related proteins including DNA-PKcs, Ku80, DNA ligase IV, and XRCC4 in the nuclear fraction compared with the cytosolic fraction was confirmed by immunoblotting (Supplementary Figure 1A). NHEJ activity was induced by addition of linearized plasmid DNA (x1 in Supplementary Figure 1B) and a resulting ladder of ligated DNA fragments (x2 and x3) was observed within 10 minutes and peaked at 30 to 60 minutes (Supplementary Figure 1B). A neutralizing antibody against DNA-PK protein blocked the observed NHEJ activity, suggesting that NHEJ activity is DNA-PK dependent (Supplementary Figure 1C). To assess inhibition of NHEJ, nuclear extracts were treated with compounds for 15 minutes and then NHEJ activity was examined. Figure 3A shows induction of NHEJ activity as the appearance of ligated DNA (x2 and x3) when linearized DNA (x1) was added in nuclear extract (lane 1 vs. 14). CC-115 inhibited NHEJ activity in a concentration-dependent manner. Partial inhibition was observed with 0.5 μM CC-115 treatment (lane 3) and 5 μM CC-115 achieved complete inhibition (lane 4). Inhibition of NHEJ was also observed in nuclear extracts treated with the known DNA-PK inhibitor NU7441 (lanes 5-7). In contrast, NHEJ activity was not changed by treatment with the mTOR kinase selective CC214-2 at concentrations of 0.05 to 5 μM (lanes 8-10). Minimal inhibition of NHEJ activity was observed with 5 μM CC214-1 treatment, consistent with its weak DNA-PK enzyme potency as compared with CC-115 or NU7441 (Supplementary Table 1). These data confirmed that inhibitors of DNA-PK, namely CC-115 and NU7441, but not mTOR kinase-selective CC214-2, inhibit NHEJ activity *in vitro*.

Inhibition of NHEJ activity by CC-115 in intact cells was tested in a cellular NHEJ assay using GFP as a reporter gene (Figure 3B and Supplementary Figure 4). Cal-51 cells treated with CC-115 (1 μM) have significantly reduced cellular NHEJ efficiency compared with DMSO-treated cells (∼21% in DMSO-treated cells versus ∼12% in 1 μM CC-115-treated cells). The results were confirmed in four separate experiments. A 384-well image-based assay was used to quantitatively measure inhibition of NHEJ by CC-115 in various cell lines. Both CC-115 and NU7441 inhibit NHEJ activity at low micro-molar concentrations while the mTOR kinase selective inhibitor CC214-2 has no effect (Table 1). The cellular potency of CC-115 in NHEJ inhibition is consistent with its cellular potency observed for pDNA-PK S2056 (Figure 1).

**Table 1:** The cellular potency of CC-115, NU7441, and CC214-2 in NHEJ inhibition

IC_50_ (μM; n=3)	CC-115	NU7441	CC214-2
**Cal-51**	3.15 ± 1.33	2.56 ± 0.84	>10
**PC3**	2.72 ± 1.69	1.03 ± 0.17	>10
**HCT116**	6.35 ± 2.98	6.22 ± 6.76	>10
**MDA-MB-231**	2.16 ± 0.91	0.63 ± 0.08	>10

IC_50_ for CC-115, NU7441 and CC214-2 of cellular NHEJ activity in Cal-51, PC3 prostate cancer, HCT116 colorectal cancer and MDA-MB-231 breast cancer lines.

### CC-115 inhibits DNA-PK and ATM activity in the reconstituted cell-free system and intact cells

To characterize the effect of CC-115 on NHEJ-related proteins in the reconstituted cell-free system, the same nuclear preparation from Figure 3A was probed by immunoblotting with antibodies against indicated NHEJ-related proteins (Figure 3C). As expected, CC-115 inhibited DNA-PK autophosphorylation at the S2056 site (pDNA-PK S2056, lanes 2-4). NU7441 (lanes 5-7), and to a smaller extent CC214-1 (lanes 11 and 12), also inhibited pDNA-PK S2056. In contrast, CC214-2 did not inhibit pDNA-PK S2056 (lanes 8-10). Total DNA-PK protein was not changed by treatment with any agent. Ku80 and XRCC4 proteins exist in fast- and slow-migrating forms, which presumably reflect differences in phosphorylation status [28]. The slow-migrating forms of Ku80 and XRCC4 (phospho-form) were induced by the presence of linear dsDNA, which mimics DNA damage (compare lanes 1 and 13), and were diminished in a concentration-dependent manner by CC-115 or NU7441 but not CC214-2 (Figure 3C, lanes 8-10). These results indicate that, as expected, CC-115 inhibits autophosphorylation of DNA-PK and the phosphorylation of the DNA-PK substrates Ku80 and XRCC4 in this reconstituted system.

CC-115 and NU7441 treatment also resulted in reduction of pATM S1981 in response to the addition of linear dsDNA (Figure 3C). To rule out the possibility that ATM is involved in NHEJ activity, nuclear extracts were treated with an ATM specific inhibitor KU55933 and the NHEJ activity was determined (Supplementary Figure 2A). KU55933 efficiently inhibited ATM phosphorylation (pATM S1981), and not DNA-PK phosphorylation (pDNA-PK S2056), in a concentration-dependent manner (Supplementary Figure 2B, lanes 5-7) but it did not inhibit NHEJ activity (Supplementary Figure 2A, lanes 5-7). This indicated that ATM activity (monitored by pATM S1981) is dispensable for NHEJ activity. Consistent with previous observations, 5 μM CC-115 or 5 μM NU7441 inhibited both DNA-PK and ATM kinase activity in this system (Supplementary Figure 2B, lanes 2 and 3).

To further evaluate the observed ATM inhibition, an additional ATM substrate, pChk2 T68, was evaluated [29]. The MCF7 nuclear extract was pretreated with CC-115, NU7441, CC214-2, or KU55933 at different concentrations (0.05, 0.5, or 5 μM) and the *in vitro* NHEJ assay was performed. As expected, concentration-dependent inhibition of pChk2 T68 was observed for the ATM kinase inhibitor KU55933 (Supplementary Figure 2C, lane 11-13). CC-115 and NU7441 also inhibited pChk2 T68 (lanes 2-4 and 5-7, respectively). Interestingly, the inhibition of both pATM and pCHK2 by CC-115 and Nu7441 was even more pronounced than that observed with KU55933.

To evaluate if observed effects on ATM activity in the nuclear extracts were recapitulated upon treatment of intact cells, HT29 colon cancer cells and MDA-MB-436 breast cancer cells were treated with CC-115 for 2 or 12 hours in the presence or absence of the DSB inducer bleomycin and the total cell lysate was used for immunoblotting (Figure 3D). Treatment with 1.5 mU/mL bleomycin strongly induced both DNA-PK and ATM activation monitored by pDNA-PK S2056 and pATM S1981, respectively. mTOR activation assessed by p4E-BP1 T37/T46 for TORC1 and pAKT S473 for TORC2 was not changed by bleomycin treatment in these two lines. When cells were treated with CC-115 in the presence of bleomycin, DNA-PK activity (pDNA-PK S2056) and ATM activity (pATM S1981 and pNbs1 S343) were significantly inhibited compared with the corresponding activities in control samples treated with DMSO or bleomycin only. Similar results were observed in Hop92 lung cancer lines (Supplementary Figures 9 and 10). These results clearly demonstrate that, as observed in the cell-free system, CC-115 inhibits both mTOR kinase and DNA-PK kinase activity in these cells under DNA damaging bleomycin treatment. In addition, although CC-115 does not directly inhibit ATM enzyme activity (IC_50_ >30 μM), and both pATM S1981 and phosphorylation of ATM substrates Chk2 and NBS1 were inhibited by CC-115 in both cell-free and intact cell systems.

ATM is required for regulation of HR induced by DNA damage. To characterize the activity of CC-115 in DNA-damage repair coupled with ATM, the effect of CC-115 on HR was evaluated. It is known that Rad51 translocates to nuclear HR sites in response to DNA damage signal and forms foci. LoVo cells were treated with 0.1 μM doxorubicin (Topoisomerase I poison, DSBs inducer) with or without CC-115 or NU7441. As expected (Supplementary Figure 3), doxorubicin strongly induces Rad51-foci in LoVo cells (24% in doxorubicin-treated cells vs 3.0% in DMSO-treated cells). CC-115 completely inhibited doxorubicin-induced Rad51-foci formation at 5 μM (3.5%). At 30 μM, inhibition below basal level of HR was observed (0.3%). NU7441 also inhibits doxorubicin-induced Rad51-foci formation but to a lesser extent (10.3%), consistent with previous observations [30–32]. Similar results were obtained in DLD1 cells as well as in a time-lapse experiment using SW480 cells expressing GFP-fused Rad51 (data not shown). These studies confirm that CC-115 not only directly inhibits DNA-PK and NHEJ but also indirectly inhibits ATM and HR induced by DNA damage in cells.

### CC-115 inhibits dissociation of NHEJ complex from DNA

**Figure 4:**
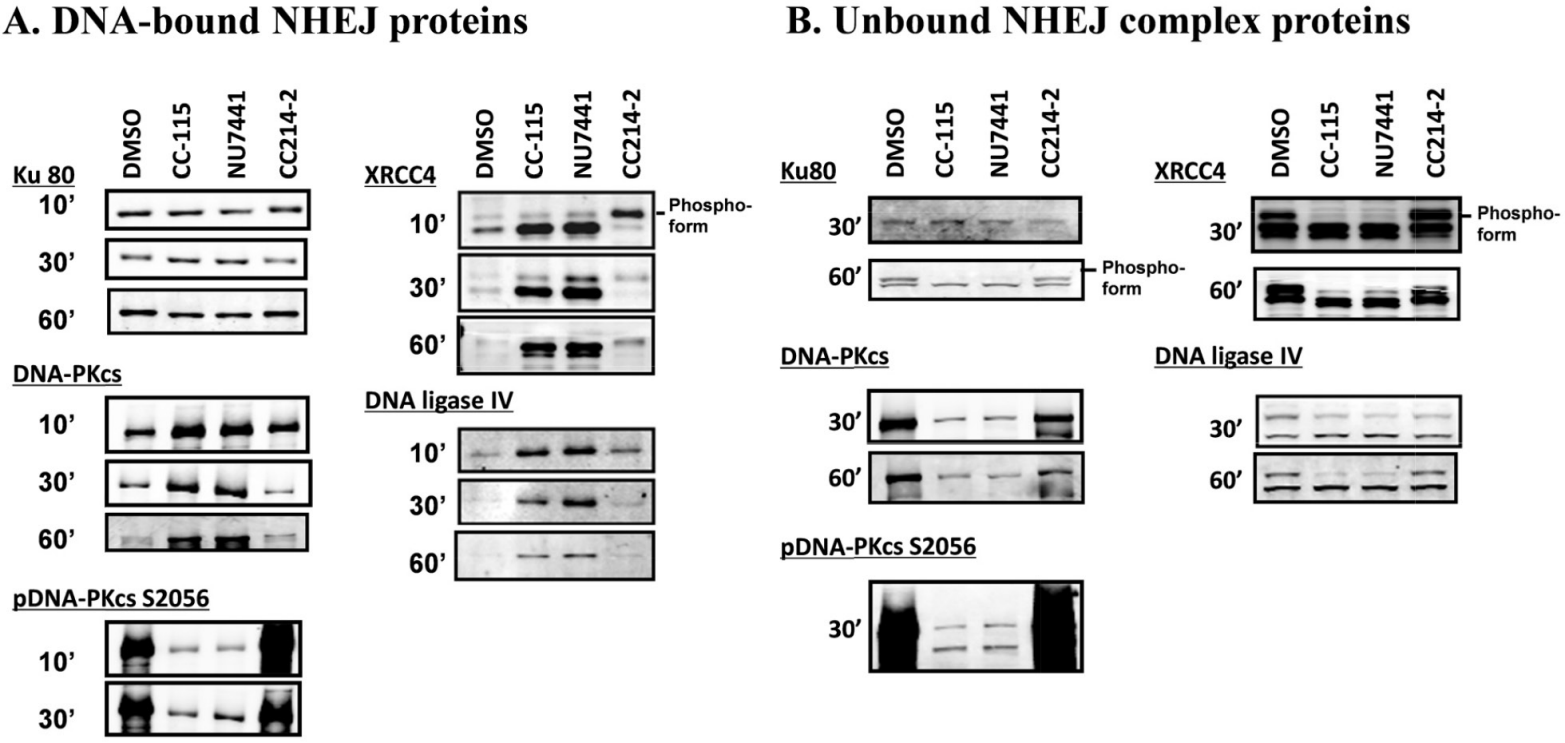
CC-115 prevents dissociation of DNA-PK, XRCC4, and DNA ligase IV from dsDNA breaks *In vitro* NHEJ assays were performed using EcoRI-digested, 5’-biotin-labeled plasmid substrate. The linearized plasmid was incubated with MCF nuclear extracts (30μg) pre-treated with DMSO or 5 μM compounds as indicated. DNA bound- **(A)** and unbound- **(B)** NHEJ complex at indicated time points were analyzed with Western blot. The experiments were independently performed at least 3 times and representative data are presented.

The stepwise assembly of the NHEJ complex (Ku80, DNA-PK, XRCC4, and DNA ligase IV) on the DNA was monitored during the NHEJ reaction *in vitro*. Biotinylated DNA was incubated with MCF7 nuclear extract pre-treated with or without CC-115, NU7441, or CC214-2 as illustrated in Supplementary Figure 5. DNA was then extracted at defined time points (10, 30, and 60 minutes) and DNA-bound NHEJ proteins were assessed by immunoblotting with the indicated antibodies (Figure 4A). In the inhibitor-free control (DMSO), Ku80 was recruited onto DNA within 10 minutes of DNA addition and remained through the end of the observed reaction (60 minutes). DNA-PKcs, XRCC4, and DNA-Ligase IV were also recruited onto the DNA within 10 minutes but were dissociated from the DNA by 30 minutes. At 60 minutes, very little DNA-PKcs, XRCC4, and DNA-Ligase IV remained on DNA. Similar results were also obtained in CC214-2-treated extracts. However, in the extract treated with CC-115 or NU7441, DNA-PKcs, XRCC4, and DNA ligase IV were retained from 10 minutes through the end of the reaction at 60 minutes. Reciprocal results were seen in analysis of the unbound fraction (Figure 4B). While the components required for NHEJ activity remain bound to DNA in the presence of DNA-PK inhibitors, NHEJ activity is completely blocked by CC-115 (Supplementary Figure 1B). These data indicate that inhibition of DNA-PK enzyme activity by CC-115 and NU7441 blocks dissociation of DNA-PKcs, XRCC4, and DNA ligase IV from damaged DNA. Western blot analysis of pDNA-PK S2056 also indicates that in the presence of CC-115 or NU7441, DNA-PK binds to DNA in its unphosphorylated form (Figure 4A).

### CC-115 preferably inhibits survival of ATM-deficient cancer cells and synergizes with DNA damaging agents in ATM-proficient cancer cells

**Figure 5:**
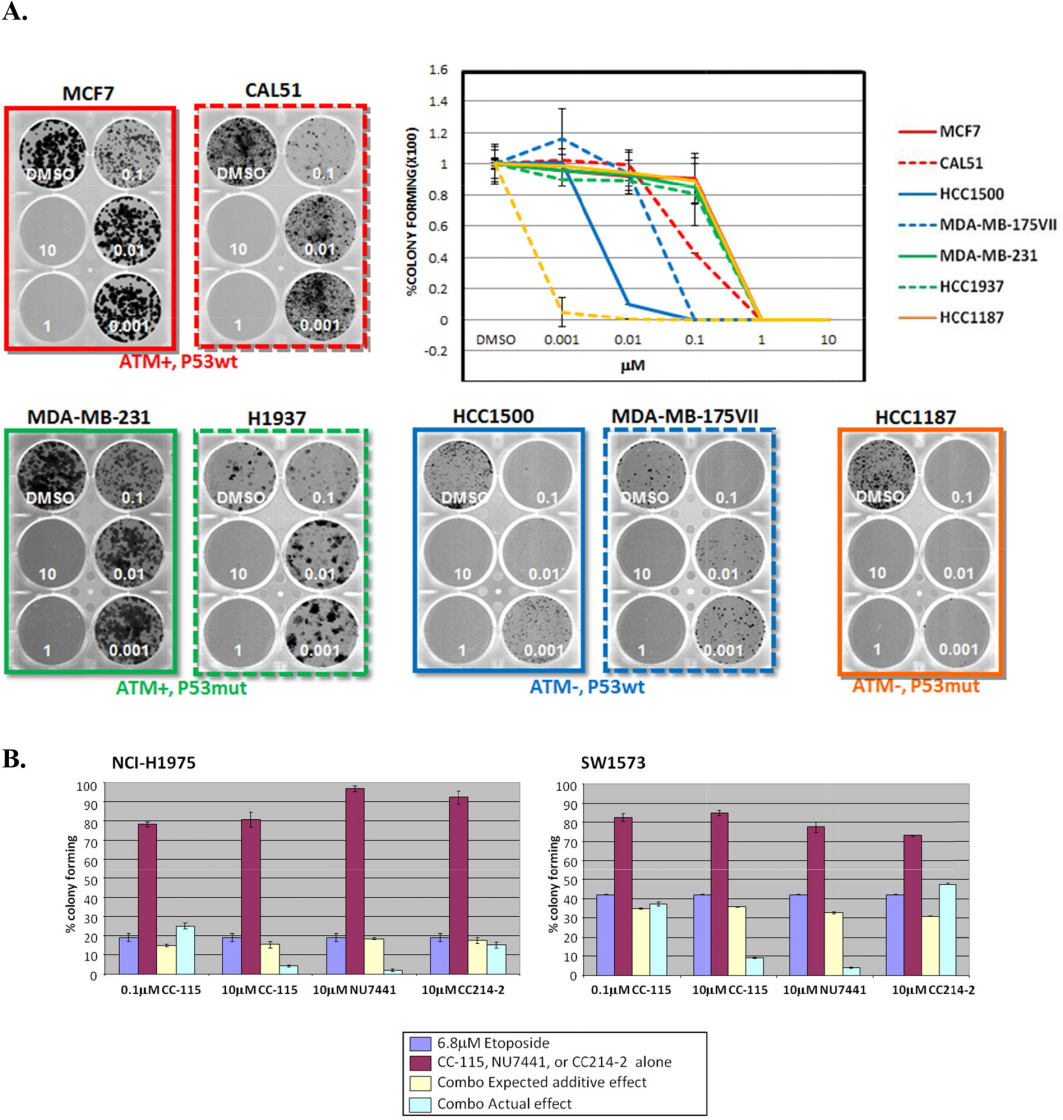
ATM-deficient cancer cells are more sensitive to CC-115 **(A)** Colony formation assays in ATM-proficient and p53-wild-type (MCF7 and CAL51, red), ATM-proficient and p53-mutant (MDA-MB-231 and H1937, green), ATM-deficient and p53-wild-type (HCC1500 and MDA-MB-175VII, blue) and ATM-deficient and p53-mutant (HCC1187, orange) cells. Cells were treated DMSO or increasing concentrations of CC-115 (0.001-10 μM), replaced with fresh media and compound every 3 days and were cultured for 2 weeks. Formed colonies were stained, photographed and counted. **(B)** CC-115 synergizes with DNA damaging agent etoposide to inhibit the survival of ATM-proficient cancer cell lines. NCI-H1975 (left) or SW1573 (right) NSCLC cells were treated with 0.1μM or 10μM CC-115, 10μM NU7441 or 10μM CC214-2 alone or together with 6.8μM etoposide for 4 hours and cultured for 2 weeks. Formed colonies were stained, photographed and counted. Expected additive effect (yellow column) was calculated by single treatment of each compound (burgundy column) and etoposide (purple column) and actual combinational effect was also shown (light blue column).

Multiple lines of evidence suggest that DNA-PK may be essential for cancer cell survival when ATM is lost [25, 33–36]. Since ATM inactivation has been reported in 20 percent of triple negative breast cancer [37], we examined a large panel of breast cancer lines for ATM and p53 levels by immunoblotting (Supplementary Figure 6). Three ATM-deficient lines (MDA-MB-175VII, HCC1187, and HCC1500) and four ATM-proficient lines (MDA-MB-231, MCF7, CAL51, and HCC1937) were selected, based on their genetic background (Supplementary Figure 7), for evaluation of CC-115 effect on survival using a colony-formation assay. The ATM-deficient lines demonstrated increased sensitivity to CC-115 for survival, with the ATM-deficient and p53-mutant line HCC1187 showing the greatest sensitivity (Figure 5A). At concentrations of CC-115 as low as 0.01 μM, colony growth is completely inhibited in HCC1187. In contrast, ATM-proficient cell lines were 100-fold less sensitive to CC-115 inhibition.

DNA-PK inhibitor NU7441 has been reported to sensitize cancer cells to radio- and chemo-induced DSBs [31]. To examine the combinatorial effect of CC-115 with DNA-damaging agents, two ATM-proficient lung cancer lines NCI-H1975 and SW1573 (See Supplementary Figure 8 for characterization of ATM status) were treated with CC-115, NU7441 or CC214-2 with or without etoposide, for 4 hours. The survival of treated cells was evaluated by colony forming capability (Figure 5B). Four-hour treatment of etoposide alone substantially impairs survival of both cell lines, while CC-115, NU7441 and CC214-2 alone have minimal effect on cell survival with this short period of treatment. Combination of 10 μM CC-115 or NU7441 but not 0.1 μM CC-115 or 10 μM CC214-2 with etoposide causes greater inhibition of colony formation than the calculated additive effect of two agents together (Figure 5B), suggesting a synergistic effect is only observed between etoposide and 10 μM CC-115 or NU7441. This observed synergy occurred only at the concentration where CC-115 caused substantial inhibition of both pDNA-PK and pATM (Supplementary Figure 9) and synergy was not observed with 10 μM of the mTOR kinase selective CC214-2. These results suggest that the inhibition of mTOR kinase does not play a significant role in the observed synergy and the inhibition of both DNA-PK and ATM may be essential for blocking cancer cell survival after DNA damage in ATM-proficient cells.

## DISCUSSION

CC-115 is a dual mTOR kinase/DNA-PK inhibitor with little activity against ATM and ATR in purified enzyme assays [26]. We report here that CC-115 inhibits both mTOR kinase and DNA-PK activity in cancer cells and potently blocks proliferation and induces apoptosis in both hematological and solid tumor cancer cell lines. The inhibition of DNA-PK by CC-115 resulted in suppression of NHEJ activity in both a reconstituted cell-free system and intact cells of multiple cancer cell lines. Consistent with others’ reports [30–32], we found that inhibition of DNA-PK by CC-115 decreases ATM activity and HR in multiple cell lines. Finally, our data indicate that CC-115 preferentially inhibits the survival of ATM-deficient cancer cell lines, suggesting patients with ATM-deficient cancers as a potential patient population for CC-115.

CC-115 inhibits DNA-PK activity, leading to inhibition of phosphorylation of DNA-PK at S2056. Phosphorylation of DNA-PK at S2056 depends on DNA-PK activity since a kinase dead DNA-PK mutant abolishes its phosphorylation in response to micro irradiation in cells [38–40]. Therefore, pDNA-PK can serve as a potential PD biomarker to monitor DNA-PK inhibition in clinical studies. We observed a large potency shift between the enzyme and cellular inhibition of DNA-PK (enzyme: 13 nM vs cell: 2600 nM). CC-115 potency against NHEJ in intact cells ranges from 1 to 7 μM in different cell lines (Table 1), consistent with its cellular potency against pDNA-PK S2056 (Figure 1). This seems to be a common phenomenon among DNA-PK inhibitors and is likely due to high endogenous levels of DNA-PK and ATP in cells.

**Figure 6:**
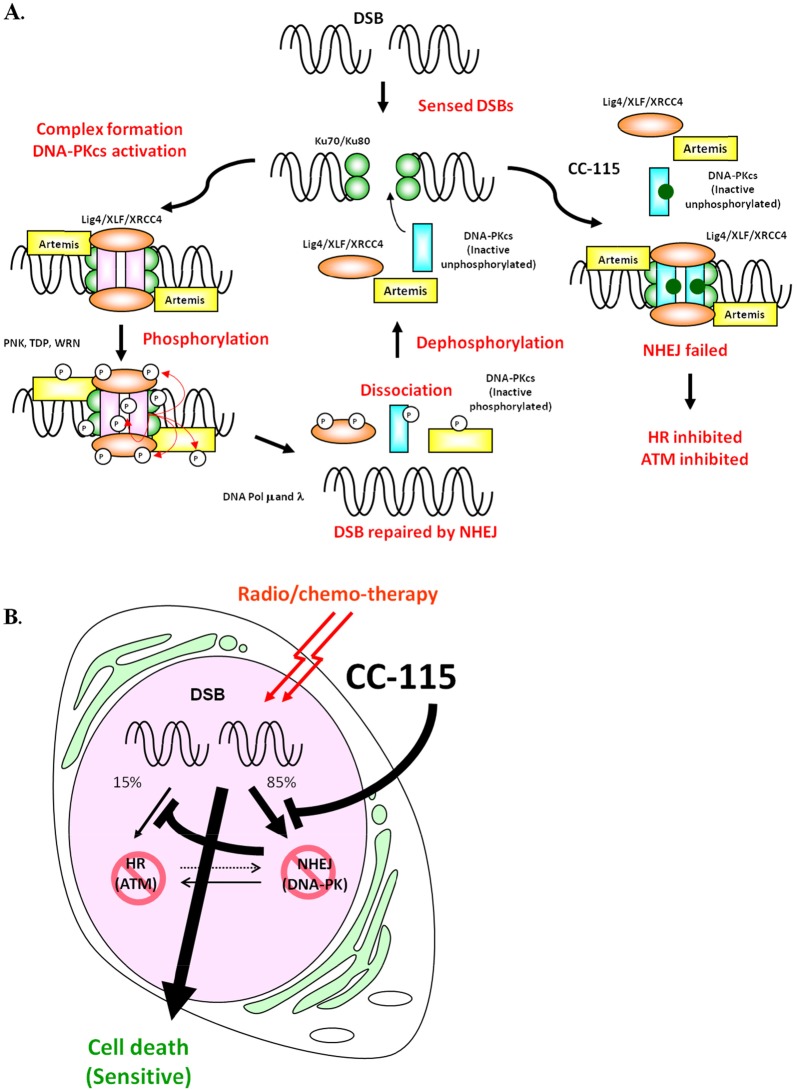
**(A)** A model illustrates how CC-115 inhibits NHEJ. DSBs are detected by Ku70/80 complex, which in turn recruits other NHEJ components including DNA-PKcs, XRCC4, XLF, DNA ligase IV, Artemis to the damage site and forms the NHEJ complex. Once DNA-PKcs binds to Ku70/80, DNA-PK is activated and phosphorylates the NHEJ complex and repairs the DNA breaks. After DSBs are repaired, NHEJ complexes dissociate from DNA and are dephosphorylated and recycled for subsequent events. In the presence of CC-115 or NU7441, DNA-PK as well as other NHEJ components (Ku80, DNA-PKcs, XRCC4 and DNA ligase IV) are recruited onto the damage site. However, inhibition of DNA-PK activity by CC-115 prevents dissociation of NHEJ components from DNA, resulting in suppression of NHEJ. **(B)** CC-115 sensitizes radio/chemo-therapy by inhibiting 2 major DSB repair pathways. CC-115 is a dual DNA-PK and mTOR kinase inhibitor that directly inhibits NHEJ by inhibiting DNA-PK and that indirectly inhibits HR by inhibiting ATM.

It was reported that autophosphorylation of DNA-PK leads to its dissociation from Ku70/80 and the DNA substrate [18, 30, 41–43]. Using a step-wise reconstituted cell-free NHEJ assay, we illustrate that DNA-PK inhibitors CC-115 and NU7441 do not prevent the association of key NHEJ factors such as Ku80, DNA-PK, XRCC4, and DNA ligase IV with dsDNA break (Figure 4A). Instead, inhibition of DNA-PK by CC-115 and NU7441 blocks dissociation of those factors from dsDNA break. Our data together with others [18, 30, 41–43] provides mechanistic insight in the role of DNA-PK activity in NHEJ as illustrated in Figure 6A. When a DSB occurs, Ku70 and Ku80 bind to the DSB and recruit unphosphorylated DNA-PK, the ligase IV/XLF/XRCC4 complex, and artemis to DNA damage sites. Complex formation with DNA and Ku70/80 activates DNA-PKcs kinase activity and results in its autophosphorylation, triggering dissociation of DNA-PK from DNA, which is essential for NHEJ. Therefore, CC-115 prevents NHEJ by blocking autophosphorylation of DNA-PKcs and dissociation of DNA-PKcs and the ligase IV/XLF/XRCC4 complex from the dsDNA end.

Although CC-115 does not directly inhibit ATM enzyme activity *in vitro*, we found that inhibition of DNA-PK by CC-115 led to suppression of ATM activity and subsequent inhibition of HR in response to dsDNA damage. It has been reported that inhibition of DNA-PK activity decreases HR in cells [30–32]. Since ATM was reported as a DNA-PK substrate, it is likely that the down-regulation of ATM activity and HR is a secondary effect of DNA-PK inhibition. Cross-regulations between ATM and DNA-PK at multiple levels have been reported previously. ATM phosphorylates DNA-PK at T2609 and regulates its activity and plays a critical role in NHEJ [18, 34, 44, 45]. DNA-PK collaborates with ATM and ATR to regulate p53-RPA interaction and facilitates HR [45]. Genetic interactions between ATM and the NHEJ factors have also been reported [46]. Knockout of both ATM and DNA-PK in mouse is embryonic lethal while mice with single knockout of each gene can survive [9, 47, 48]. Based on the cross interaction between ATM and DNA-PK, it has been proposed that repression of NHEJ may lead to cytotoxicity against ATM-deficient cells while sparing healthy cells [25, 49]. Consistent with this hypothesis, we found that ATM-deficient cancer lines are more sensitive to CC-115 than ATM proficient lines, suggesting patients with ATM-deficient tumors such as breast cancer as potential patient populations for clinical development of CC-115. NHEJ is a key determinant of chemo- and radio-resistance and loss of DNA-PK sensitizes cells to DNA damaging agents [17]. We show that CC-115 sensitized two ATM-proficient cancer cell lines to etoposide treatment, suggesting that inhibition of dsDNA repair pathways by CC-115 may also sensitize tumor to radio- and chemo-therapy that induce DNA damage (Figure 6B).

In conclusion, we demonstrated that CC-115, a potent inhibitor of mTOR kinase and DNA-PK, inhibits mTOR kinase and DNA-PK activity in cell-free and in cellular systems. Under DNA damaging conditions, inhibition of DNA-PK signaling by CC-115 results in suppression of DNA repair by both the NHEJ and HR pathways. These studies support the use of CC-115 in combination with DNA damaging agents in the clinic. Finally, the proposed synthetic lethality between the ATM and DNA-PK pathways and the activity observed in this study suggests the use of ATM deficiency as a patient selection strategy for CC-115 as a single agent.

## MATERIALS AND METHODS

### Cell lines, compounds and antibodies

Cell lines were purchased from American Tissue Culture Collection of Microorganism and Cell Culture. Cells were cultured in growth media as recommended by the vendor. Upon receipt, each line was expanded and frozen down at low passage. Cells were then used for limited passages of no more than 2 months. CC-115 (1-ethyl-7-(2-methyl-6-(1*H*-1,2,4-triazol-3-yl)pyridin-3yl)-3,4-dihydropyrazino[2,3-b]pyrazin-2(1*H*)-one) was identified following a screening campaign and compound optimization efforts [26]. CC214-1 (6-(4-(1*H*-1,2,4-triazol-3-yl)phenyl)-1-(2-(tetrahydro-2*H*-pyran-4-yl)ethyl)-1,3-dihydro-2*H*-imidazo[4,5-b]pyrazin-2-one) and CC214-2 (6-(6-(2-hydroxypropan-2-yl)pyridin-3-yl)-4-((tetrahydro-2*H*-pyran-4-yl)methyl)-3,4-dihydropyrazino[2,3-b]pyrazin-2(1*H*)-one) are mTOR kinase inhibitors [50]. DNA-PK inhibitor NU7441 (8-(4-dibenzothienyl)-2-(4-morpholinyl)-4H-1-benzopyran-4-one, TOCRIS, Ellisville, MO), ATM inhibitor KU55933 (2-(4-morpholinyl)-6-(1-thianthrenyl)-4H-pyran-4-one, TOCRIS, Ellisville, MO), DNA damaging reagents bleomycin (VWR, Pittsburg, PA), and doxorubicin (Sigma, St Louis, MO) were purchased from the indicated sources. Antibodies for pDNA-PKcs S2056 (ab18192), pATM S1981 (ab81292), Ku80 (ab3107), XRCC4 (ab145), DNA ligase IV (ab80514), and Rad51 (ab213) were purchased from Abcam (Cambridge, MA). Antibodies for S6 (2317), pS6 S235/236 (4857), p4EBP1 T46/37 (2855), pNbs1 S343 (3001) and pChk2 T68 (2661) were purchased from Cell Signaling Technology (Denver, CO). Antibodies for DNA-PKcs (Clone 18-2), 4EBP1 (sc-6025), ATM (Ab-3), and ATM pS1981 (10H11.E12) were purchased from Thermo (Fremont, CA), Santa Cruz Biotechnology (Santa Cruz, CA), Calbiochem (San Diego, CA), and Rockland (Gilbertsville, PA), respectively.

### Determination of cellular proliferation and apoptosis.

Proliferation assays were performed as previously described [51]. For apoptosis measurement, cells were treated with increasing concentrations of CC-115 for 24 h and then assessed for Caspase 3/7 induction compared to DMSO control using Caspase 3/7-Glo (Promega). The concentration of compound required for a 2-fold induction of Caspase was determined (CalX).

### Reconstituted cell-free and cellular NHEJ assays

MCF7 human breast cancer cells (5-10 x 10^7^ each) were incubated in 5 pellet volumes of cold hypotonic buffer (10 mM Hepes-KOH, pH 7.5, 1.5 mM MgCl_2_, 5 mM KCl, and 0.5 mM DTT) for 20 minutes and were homogenized in a tight-fitting glass dounce homogenizer for 10-20 strokes. The nuclei were then collected by low-speed centrifugation and nuclear proteins were extracted by high-salt buffer (50 mM Tris-HCl, pH 7.5, 1 M KCl, 2 mM EDTA, 1 mM DTT, and protease inhibitors). The extracted nuclear proteins were dialyzed against 1 L of dialysis buffer (20 mM Tris-HCl, pH 8.0, 20% glycerol, 0.1 M KOAc, 0.5 mM EDTA, and 1 mM DTT) for 1 hour and were used for the *in vitro* NHEJ assay.

Nuclear protein (10-20 μg) prepared above was pre-treated with compounds as indicated on ice for 15 minutes. Twenty-five nanograms of EcoRI-digested pBluescript DNA (Stratagene, La Jolla, CA) were then added and the mixture was adjusted to 20 μL final volume in NHEJ buffer (50 mM Hepes-KOH pH 8.0, 40 mM KOAc, 1 mM Mg(OAc)_2_, 1 mM ATP, 0.1 mg/mL BSA, and 1 mM DTT). After incubation at 25°C for 60 minutes, the reaction was terminated by incubation with 1 μg/mL RNase A for 10 minutes followed by treatment with 2 mg/mL Protease K in 0.5% SDS at 55°C for 30 minutes. The reactions were run onto 0.8% agarose and the gel was stained with Sybr Green (S756, Invitrogen, Carlsbad, CA). For Western blots, an aliquot of the same reaction mixture described above was subjected to SDS-PAGE, transferred to the PVDF membrane, and immunoblotted with indicated antibodies.

Details for the cellular NHEJ assay is described in Supplementary Methods.

### DNA end binding assay

One kb of DNA fragment, including an EcoRI sequence in the middle, was amplified by PCR using pEGFP as a template and a pair of biotinylated EGFP primers (5′-biotin-CTTCAAGTCCGCCATGCCCG-3′ and 5′-biotin-GGCCATCGCCCTGATAGACG-3′: Valuegene, San Diego, CA). The amplified DNA fragment was cleaved by EcoRI to produce two 0.5 kb biotinylated DNA fragments, and was purified using the QIAquick PCR purification kit (Qiagen). Five pmol of the biotinylated DNA fragment were immobilized on 10 μL of Dynabeads MyOne Streptavidin T1 paramagnetic beads (catalog #65601, Invitrogen, Carlsbad, CA) in 50 μL of binding buffer (5 mM Tris-HCl, pH7.5, 0.5 mM EDTA, and 1 M NaCl) and DNA- or mock-treated beads were incubated in 25 μL of reaction mixture containing 30 μg of nuclear extract in reaction buffer (40 mM Hepes-KOH, pH 8.0, 5 mM MgCl_2_, 60 mM KCl, 0.5 mM DTT, 0.5 mM EDTA, 3.4% glycerol, and 0.3 mg/mL BSA) at 25°C for 10, 30, or 60 minutes. An aliquot of the reaction was removed at each time point to separate the supernatant and the beads. The supernatant was subjected to SDS-PAGE and immunoblotting with indicated antibodies. The beads were washed twice with 250 μL of IP buffer (25 mM Hepes-KOH, pH 7.5, 100 mM NaCl, 20% glycerol, 5 mM EDTA, 1 mM DTT, 0.05% Nonidet P-40, 10 mM NaF, and protease inhibitor mixture) and subjected to SDS-PAGE and immunoblotting with indicated antibodies.

### Immunoblotting

Cells were treated with 5 μM CC-115 with or without 1.5 mU/mL bleomycin for the indicated time and lysed in RIPA buffer (50 mM Tris-HCl, pH 7.4, 150 mM NaCl, 0.25% deoxycholic acid, 1% Nonidet P-40, 1 mM EDTA, and protease inhibitors). The cell lysates were subjected to SDS-PAGE and immunoblotting with indicated antibodies. NCI-H441 human lung cancer cells were treated with indicated concentrations of CC-115 with or without 1.5 mU/mL bleomycin for 2 hours and pDNA-PK in the cell lysates was measured using immunoblotting. The pDNA-PK bands were quantified by Odyssey imaging software (LI-COR, Lincoln, NE) and normalized to total DNA-PK. IC_50_ values were determined by Excelfit (London, UK).

### Clonogenic assay

MCF7, Cal51, MDA-MB-231, H1937, MDA-MB-175VII and HCC1187 human breast cancer cell lines were plated at 2000 cells per well in 6-well plates and HCC1500 breast cancer cell line was plated at 10,000 cells per well in 6-well plates and cultured overnight. Cells were then treated with compounds at indicated concentrations. Media and compounds were changed every three days. Plates were incubated for a total of about two weeks then fixed and stained using the DIFF QUIK staining kit (Microptic, Barcelona, Spain). Colonies were imaged and counted.

NCI-H1975 and SW1573 human lung cancer cell lines were treated with compounds (CC-115, NU7441 or CC214-2 together with 6.8 μM Etoposide) at 0.1 or 10 μM at 37°C for four hours and subsequently plated at 5,000 cells per 10cm dish with fresh media. Media were changed every three days. Plates were incubated for a total of about two weeks then fixed and stained using the DIFF QUIK staining kit. Colonies were imaged and counted.

## SUPPLEMENTARY MATERIALS TABLES AND FIGURES





## Abbreviations

NHEJ: non-homologous end joining
HR: homologous recombination
PD: pharmacodynamic
mTOR: mammalian target of rapamycin
PIKK: phosphoinositide 3-kinase related kinase
DNA-PK: DNA-dependent protein kinase
DNA-PKcs: catalytic subunit of; DNA-dependent protein kinase
SMG1: suppressor of morphogenesis in genitalia
TRRAP: transformation / transcription domain-associated protein
Rapalogs: Rapamycin analogs
IRS1: insulin receptor substrate 1
RCC: renal cell carcinoma
dsDNA: double-strand DNA
DSBs: dsDNA breaks
