# Supplementary material for: CC-115, a dual inhibitor of mTOR kinase and DNA-PK, blocks DNA damage repair pathways and selectively inhibits ATM-deficient cell growth in vitro
Source: Oncotarget. 2017 Aug 18;8(43):74688–702. doi: 10.18632/oncotarget.20342 (PMC5650372; doi:10.18632/oncotarget.20342)
Supplement: Supplementary file 1 [file oncotarget-08-74688-s001.pdf]

CC-115, a dual inhibitor of mTOR kinase and DNA-PK, blocks DNA damage repair pathways and selectively inhibits ATM-deficient cell growth *in vitro*

SUPPLEMENTARY MATERIALS

Kinase assays

mTOR, DNA-PK and PI3K-alpha assays were performed as reported previously [1, 2].

ATM and ATR assay and IC_{50} determination

HA-ATM and FLAG-ATR (HA-labeled ATM, and FLAG-labeled ATR) were each transiently expressed in HEK293 cells (293FT cells, Invitrogen Cat: R700-07) using Lipofectamine as transfection reagent. Cells were harvested 6 days post transfection and spun down to separate cells from media. Cells were then lysed using a lysis buffer (50 mM Tris buffer, (Tris(hydroxylmethyl)-aminomethane, pH 7.5), 100 mM NaCl, 50 mM βGOP, 10% Glycerol, 0.5% Tween 20, 1 mM EDTA, 25 mM sodium fluoride (NaF), 20 nM microcystine, 2 mM phenylmethylsulfonylfluoride (PMSF) and protease inhibitor cocktail) followed by sonication 3 times and a final spin at 20,000 rpm for 30 minutes to generate a clear lysate. To perform the immunoprecipitation, enzymes were captured on Protein G Sepharose from GE Healthcare (Cat. No. 17-0618-01) using specific antibodies to the HA or FLAG tags. Beads plus enzymes were then washed 3 times with lysis buffer and spun down at 1000 rpm for 1 minute. Beads plus enzymes were resuspended in lysis buffer and kept at 4°C until assay. Assays for ATM and ATR were conducted in a ^{33}P radioactive assay format. Assay buffer contained the following reagents: 1 mg/mL GST-p53 (glutathione S-transferase–p53 fusion protein) substrate (expressed in-house), 20 µg/mL calf thymus DNA activator (Sigma), 100 µg/mL BSA, 200 µM EGTA (ethylene glycol tetraacetic acid), 100 µM EDTA, and 1 mM DTT. Reactions were run in a 100-µL assay volume. The ATP concentration used for all 3 assays was 100 µM with 3 µCi of ^{33}P-labeled ATP per well (Perkin-Elmer Catalog No. NEG602H001MC). Reaction times were 3.5 hours for ATM and 4 hours for ATR. Reactions were stopped with 100 µL of 3% phosphoric acid. Samples were then transferred to a filter plate and washed with 0.75% phosphoric acid using a vacuum manifold. After allowing wells to dry for 15 minutes, 50 µL of scintillation fluid were added to each well, and the plate was read using a Topcount reader. Enzyme inhibition by CC-115 was tested from 2 separate dispenses in triplicate at 2 concentrations (3 and 30 µM). The total radioactivity incorporated in the presence of CC-115 was compared with the radioactivity incorporated in the corresponding no inhibitor control to determine percent inhibition. An IC_{50} value was then estimated.

Cellular NHEJ assay

The experimental design is illustrated in Supplementary Figure 4. Cal-51 breast cancer cells were separately transfected with pEGFP plasmid (6085-1, Clontech, Mountain View, CA) digested with either EcoRI (to measure NHEJ) or StuI (to measure transfection efficiency) using a Amaxa cell line nuclearfector kit V (CCA-1003, Lonza). Immediately after the transfection, the cells were treated with DMSO or compounds at indicated concentrations for 24 hours and GFP-positive cells were counted under a fluorescent microscope. NHEJ activity was calculated by the number of GFP-positive cells in cells transfected with pEGFP-EcoRI divided by that in cells transfected with pEGFP-StuI, multiplied by 100. To quantitatively determine the potency of CC-115 and other compounds in inhibition of NHEJ activity in multiple cell lines, a higher throughput NHEJ assay was developed. Different cell lines were transfected using the procedure described above and subsequently seeded into 384-well plates at optimized density for individual cell lines. Cells were then treated with compounds at increasing concentrations. GFP+ cells were imaged and quantitated using the IncuCyte (Essen Bioscience, Ann Arbor, MI). NHEJ activity was then normalized to the DMSO control and fitted to the Sigmoidal Dose-Response Model (IDBS XLFit Model 205; London, UK) to determine the activity of each compound. Results were expressed as a IC_{50} value, which is the compound concentration required to inhibit 50% of GFP-positive cells during the 24 hours of treatment compared to the DMSO control. The IC_{50} value was calculated using the following equation: y = (A+ ((B-A) / (1 + ((C/x)^D)))) where $A = Y_{Min,}$ $B = Y_{Max}$, $C = EC_{50}$, D = Hill Slope, y = Relative NHEJ activity, and the IC_{50} is the concentration of the compound when y = 50% of DMSO control.

Immunofluorescence

DLD1 or LoVo human colorectal cancer cell lines cultured on coverslips were extracted with Cytoskeleton

(CSK) buffer (0.5% Triton-X100, 10 mM Hepes, pH 7.4, 100 mM NaCl, 300 mM sucrose, 3 mM $MgCl_2$, 50 mM NaF, and 0.1 mM $NaVO_3$) on ice for 5 minutes to remove cytosolic and nucleosolic proteins but retain the chromatin-bound proteins, fixed with 4% phosphate buffered paraformaldehyde solution for 10 minutes, neutralized with 0.1 M glycine for 5 minutes, and incubated in blocking buffer (0.4% Triton X-100, 2% fetus bovine serum in PBS) for 20 minutes at room temperature. The cells were incubated with mouse anti-Rad51 antibody, washed with washing buffer (0.2% Triton X-100 and 0.2% BSA in PBS), and then incubated with Alexa Fluor594-conjugated goat anti-mouse secondary antibody (invitrogen, Carlsbad, CA) at room temperature for 1 hour. After washing, the cells were mounted in ProLong Antifade mounting solution (invitrogen, Carlsbad, CA) with 4′,6-diamidino-2-phenylindole (DAPI) and sealed. The slides were imaged and photographed using a fluorescent microscope. All images were processed using Adobe Photoshop for presentation. To monitor inhibition of HR by CC-115, at least 600 nuclei from each slide were counted and HR efficiency was calculated by number of the nuclei containing more than 10 foci (HR positive) per number of the total nuclei.

REFERENCES

1. Mortensen DS, Fultz KE, Xu S, Xu W, Packard G, Khambatta G, Gamez JC, Leisten J, Zhao J, Apuy J, Ghoreishi K, Hickman M, Narla RK, et al. CC-223, a potent and selective inhibitor of mTOR kinase: *in vitro* and *in vivo* characterization. Mol Cancer Ther. 2015; 14:1295-1305.
2. Mortensen DS, Perrin-Ninkovic SM, Shevlin G, Elsner J, Zhao J, Whitefield B, Tehrani L, Sapienza J, Riggs JR, Parnes JS, Papa P, Packard G, Lee BG, et al. Optimization of a series of triazole containing mammalian target of rapamycin (mTOR) kinase inhibitors and the discovery of CC-115. J Med Chem. 2015; 58:5599-5608.
3. Leahy JJ, Golding BT, Griffin RJ, Hardcastle IR, Richardson C, Rigoreau L, Smith GC. Identification of a highly potent and selective DNA-dependent protein kinase (DNA-PK) inhibitor (NU7441) by screening of chromenone libraries. Bioorg Med Chem Lett. 2004; 14:6083-6087.
4. Mortensen DS, Sapienza J, Lee BG, Perrin-Ninkovic SM, Harris R, Shevlin G, Parnes JS, Whitefield B, Hickman M, Khambatta G, Bisonette RR, Peng S, Gamez JC, et al. Use of core modification in the discovery of CC214-2, an orally available, selective inhibitor of mTOR kinase. Bioorg Med Chem Lett. 2013; 23:1588-1594.

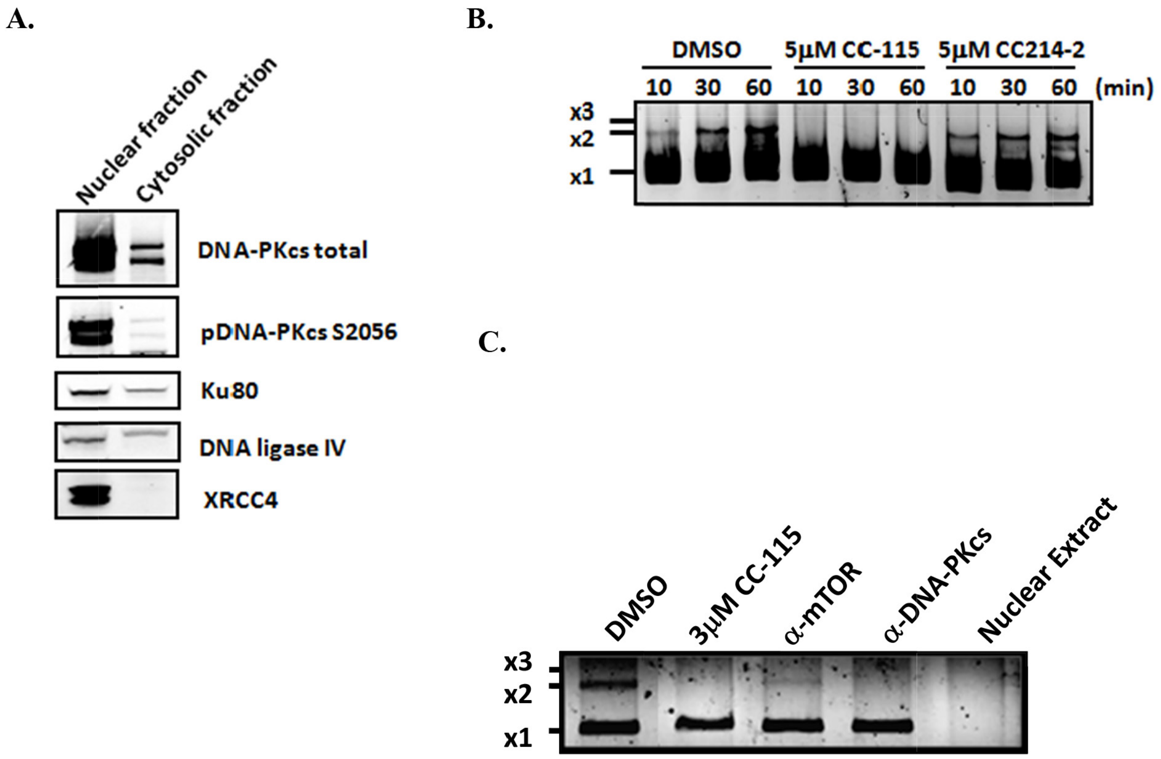

Supplementary Figure 1: (A) Western blot analysis of subcellular fractionation of MCF7 breast cancer cells. **(B** and **C)** Inhibition of NHEJ by CC-115 (B) or anti-DNA-PK antibody (C). Linearized plasmid DNA was incubated with nuclear extract pre-treated with indicated compounds or antibodies in an *in vitro* NHEJ assay.

A. NHEJ Assay

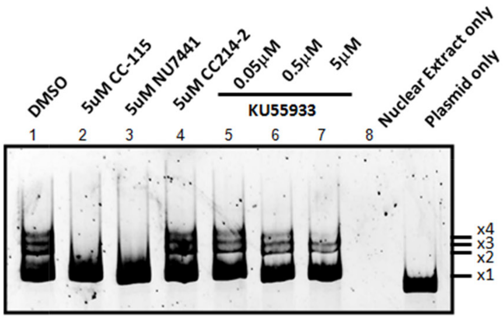

B. Western Blot

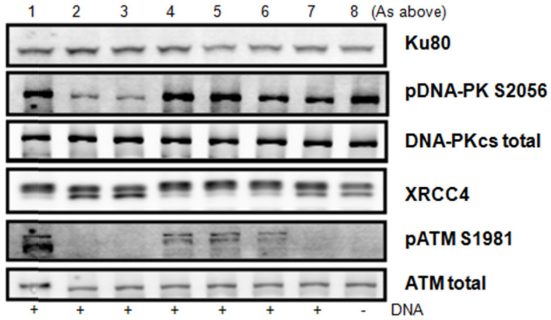

C. Western Blot (nuclear extract from NHEJ assay)

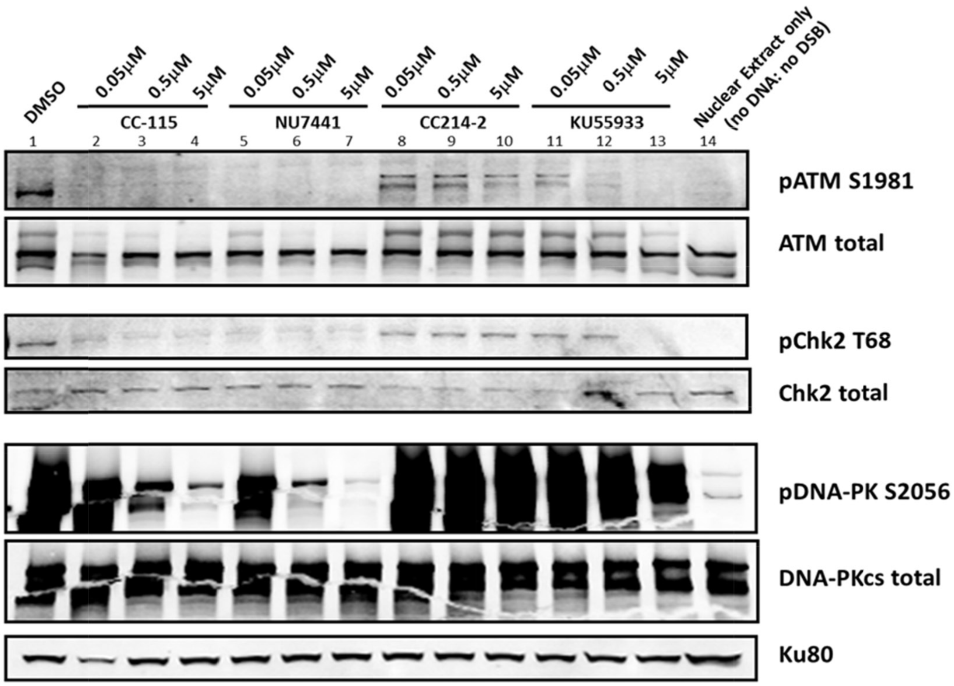

Supplementary Figure 2: ATM activity is not required for NHEJ. **(A)** Ligation of linearized DNA fragments by NHEJ. Linearized plasmid DNA was incubated with MCF nuclear extracts pre-treated with DMSO or compounds in an *in vitro* NHEJ assay. **(B)** Western blot of nuclear extracts from (A). **(C)** CC-115 inhibits ATM activity. Western blot analysis of MCF nuclear extracts pre-treated with DMSO or different compounds in presence or absent of linearized plasmid DNA.

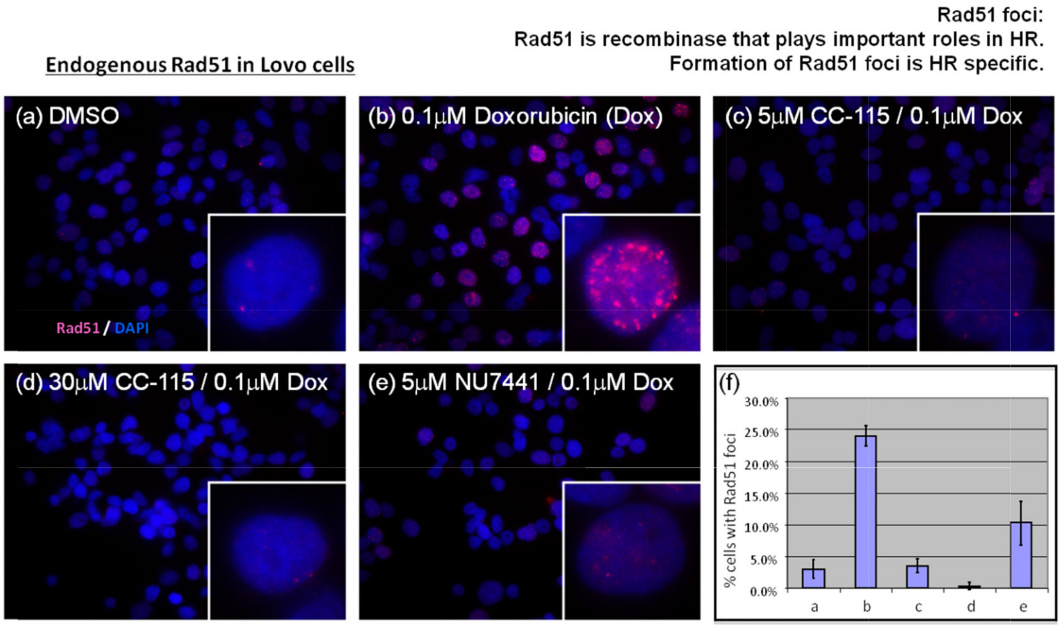

Supplementary Figure 3: CC-115 inhibits homologous recombination in cells. Lovo colorectal cancer cells were cultured on coverslips and were then treated with **(A)** DMSO, **(B)** 0.1µM doxorubicin only, combination of 0.1µM doxorubicin and **(C)** 5µM or **(D)** 30µM CC-115 or **(E)** 5µM NU7441. Cells were extracted with CSK buffer to remove cytosolic and nucleosolic proteins but retain the chromatin-bound proteins and were stained with anti-Rad51 antibody and DAPI and were photographed under microscope. At least 600 nuclei from each slide were counted and nuclei containing more than 10 foci were classified as HR positive.

GFP-Based NHEJ Assay

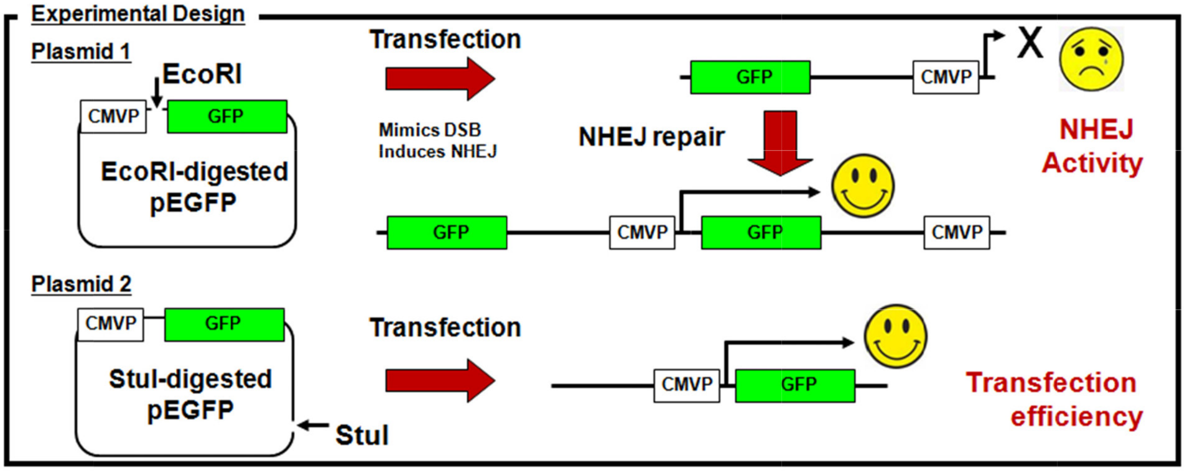

Supplementary Figure 4: Experimental design of NHEJ reporter assay.

Experimental Design

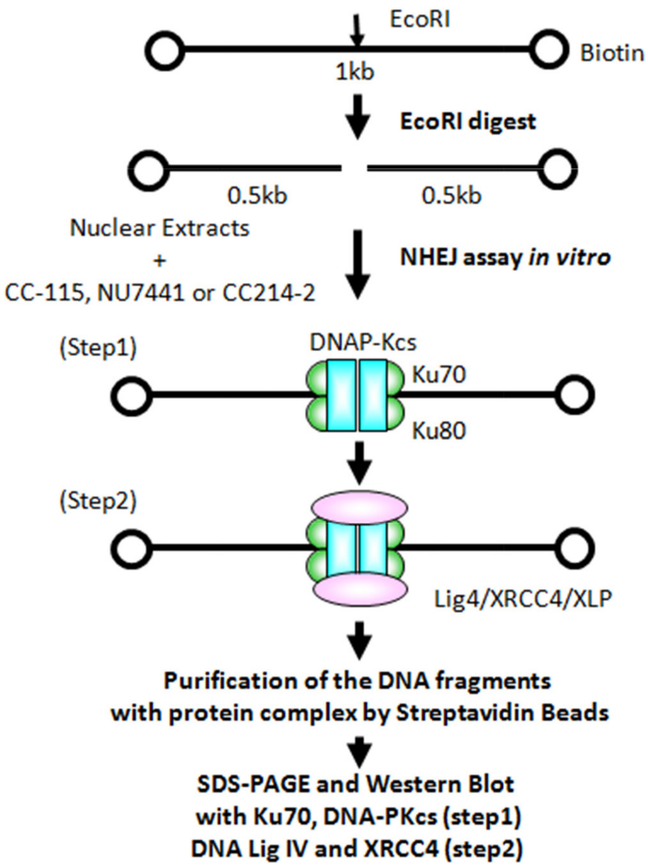

Supplementary Figure 5: Experimental design of DNA end binding assay.

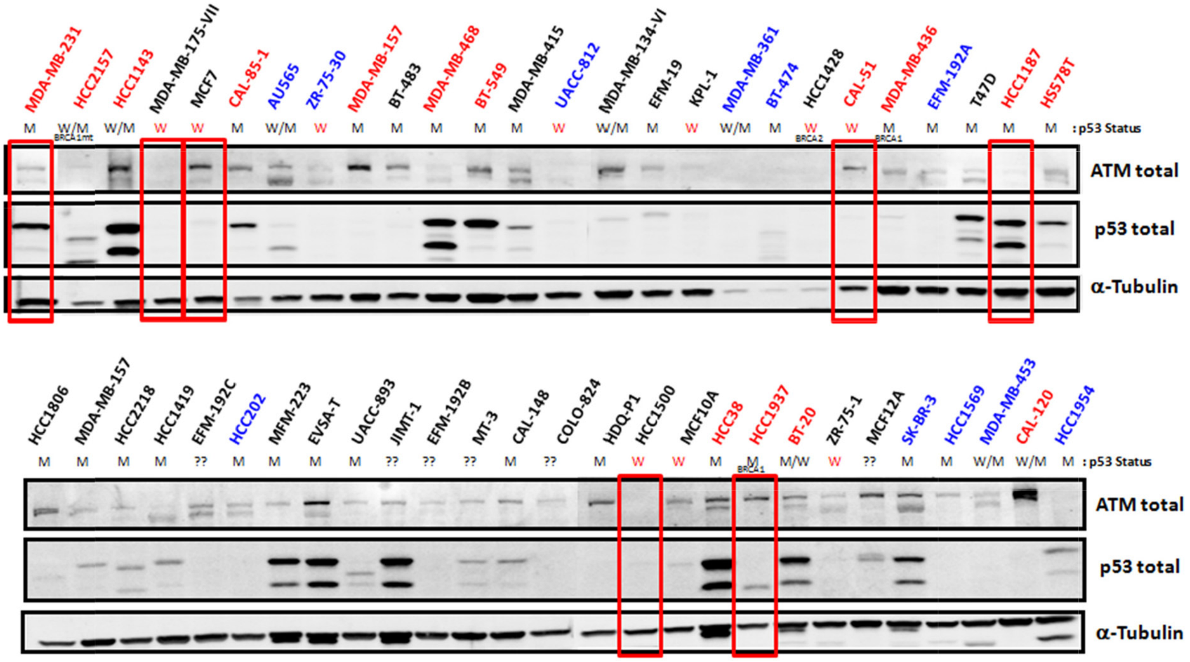

Supplementary Figure 6: Expressions of ATM and p53 in human breast cancer cell lines. Expression levels of ATM and p53 were examined in 53 human breast cancer cell lines. Molecular subtypes of breast cancer: Luminal A/B **(ER+PR+)**, HER2 type **(HER2+)** and Triple negative/basal-like **(TN)** were shown in **black**, **blue** and **red**, respectively. TP53 gene status were also shown as W (wild-type) and M (mutant-type). Cell lines that used for clonogenic assay were shown in red rectangle.

CELL LINE	Concensus subtype	Subtype	Notes	APC	BRAF	CDKN2A gene	KRAS	PIK3CA consensus	PTEN consensus	Rb gene consensus	STK11	TP53 gene Consensus	CDKN2A consensus	BRCA1	BRCA2	ATM (expression levels)
(1) ATM+/p53wt																
MCF7	Luminal	ER+/PR+	WNT7B+	WT	WT	M1 *157del or G6E	WT	E545K	pos	WT	WT	WT	mut	WT	WT	High
CAL-51	Basal	TN			WT	WT	WT	E542K	neg	WT		WT	WT			Middle
(2) ATM+/p53mt																
MDA-MB-231	Basal	TN		WT	G464V	M1 *157del or WT	G13D	WT	pos	WT	WT	R280K	mut	WT	WT	Middle
HCC1937	Basal	TN	BRCA1 mutated	WT	WT	WT	WT	WT	neg	K715* or WT	WT	R306*	WT	mut	WT	Middle
(3) ATM-/p53wt																
MDA-MB-175-VII	Luminal	ER+/PR+	WNT7B+	WT	WT	WT	WT	WT	pos	WT	WT	WT	WT	WT	WT	Low
HCC1500	Luminal	ER+/PR+			WT	WT	WT	WT	pos	WT		WT	mut			Low
(4) ATM-/p53mt																
HCC1187	Basal	TN		WT	WT	WT	WT	WT	pos	WT	WT	G108del	WT	WT	WT	Low

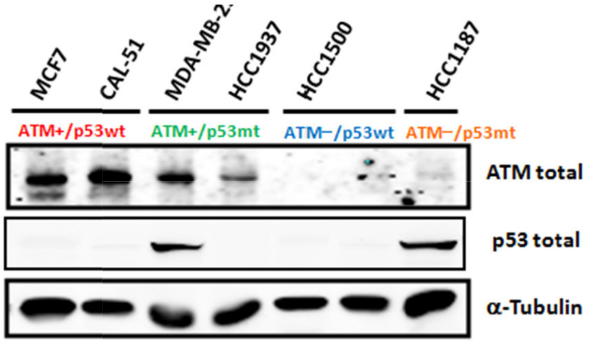

Supplementary Figure 7: Genetic information of cell lines used for clonogenic assay. Top: Genetic information of human breast cancer cell lines used for clonogenic assay were shown. Bottom: Expressions of ATM and p53 were examined in cancer cell lines used for clonogenic assay.

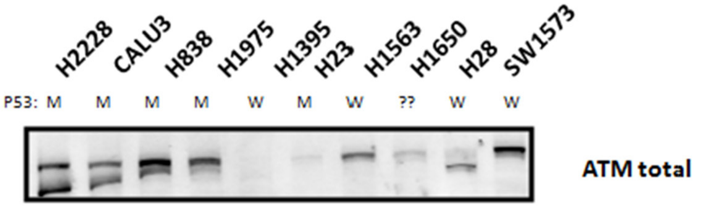

CellLine	Tissue	PIK3CA	TP53	CDKN2A	KRAS	EGFR	RB1	STK11	PTEN
NCI-H1975	Lung	p.G118D	p.R273H	p.E69*	wt	p.T790M-p.L858R	wt	wt	wt
SW1573	Lung	p.K111E	wt	p.M1_*157del	p.G12C	wt	wt	wt	wt

Supplementary Figure 8: Expression level of ATM in human NSCLC cell lines. Top: Expression of ATM were examined in 10 human NSCLC cell lines. P53 gene status were also shown in W (wild-type) and M (mutant-type). Bottom: Genetic information of human lung cancer cell lines used for clonogenic assay were shown.

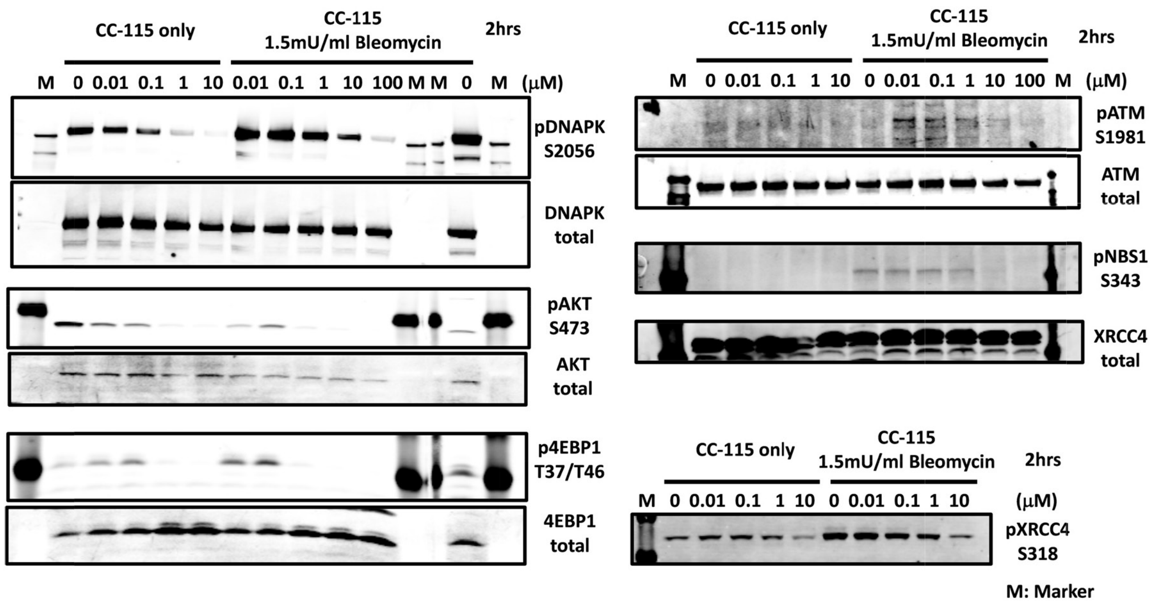

Supplementary Figure 9: CC-115 inhibits ATM activity in ATM-proficient Hop92 cells. Western blot analysis of ATM-proficient HOP92 cells treated with CC-115 as indicated with or without 1.5mU/ml Bleomycin for 2 hours.

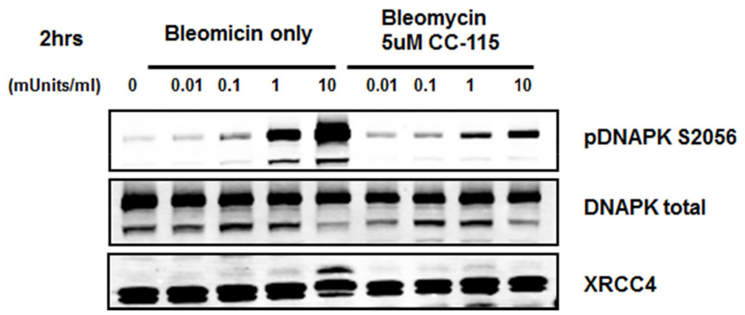

Supplementary Figure 10: Dose-dependent activation of DNA-PK by bleomycin in Hop92 cells.

Supplementary Table 1: IC_{50} of compounds used in this study

	mTOR kinase (IC_{50} µM)	DNA-PK (IC_{50} µM)	ATM (IC_{50} µM)	ATR (IC_{50} µM)
**CC-115 [2]**	0.021 ± 0.002	0.013 ± 0.001	>30	>30
**NU7441 [3]**	1.64 ± 0.20	0.015 ± 0.20	>100*	>100*
**CC214-2 [4]**	0.106 ± 0.026	>10	NT	>3
**CC214-1 [4]**	0.002 ± 0.0002	0.141^	NT	NT

NT: not tested, *Lit. reported values [3], ^ n=2.
Compound potency against mTOR kinase, DNA-PK, ATM and ATR. Average IC_{50} values (± SEM) in µM are listed.

Supplementary Table 2: Cellular growth inhibition for CC-223 across a panel of cancer cell lines

See Supplementary File 1
