# Supplementary material for: CC-115, a dual inhibitor of mTOR kinase and DNA-PK, blocks DNA damage repair pathways and selectively inhibits ATM-deficient cell growth in vitro
Source: Oncotarget. 2017 Aug 18;8(43):74688–702. doi: 10.18632/oncotarget.20342 (PMC5650372; doi:10.18632/oncotarget.20342)
Supplement: Supplementary file 2 [file oncotarget-08-74688-s002.docx]

**Supplementary Table 2.** Cellular growth inhibition for CC-223 across a panel of cancer cell lines.

| **Cell line** | **Disease** | **Tumor type** | **Tumor subtype** | **n** | **GI50** | **GI50 SEM** | **IC50** | **IC50 SEM** |
| --- | --- | --- | --- | --- | --- | --- | --- | --- |
| KARPAS-422 | Hematological | NHL | DLBCL | 5 | 0.015 | 0.004 | 0.024 | 0.005 |
| RIVA | Hematological | NHL | DLBCL | 3 | 0.016 | 0.006 | 0.096 | 0.028 |
| KASUMI-1 | Hematological | Leukemia | M-AML | 3 | 0.041 | 0.011 | 0.118 | 0.018 |
| KG-1 | Hematological | Leukemia | M-AML | 3 | 0.046 | 0.005 | 0.393 | 0.040 |
| WSU-NHL | Hematological | NHL | FL | 3 | 0.047 | 0.002 | 0.066 | 0.002 |
| JEKO-1 | Hematological | NHL | MCL | 3 | 0.066 | 0.022 | 0.097 | 0.027 |
| Toledo | Hematological | NHL | DLBCL | 3 | 0.072 | 0.010 | 0.441 | 0.010 |
| KARPAS-1106P | Hematological | NHL | DLBCL | 3 | 0.072 | 0.015 | 0.074 | 0.015 |
| NU-DHL-1 | Hematological | NHL | DLBCL | 4 | 0.080 | 0.017 | 0.111 | 0.027 |
| RC-K8 | Hematological | NHL | DLBCL | 3 | 0.082 | 0.008 | 0.145 | 0.015 |
| SU-DHL-8 | Hematological | NHL | DLBCL | 3 | 0.084 | 0.023 | 0.099 | 0.024 |
| Pfeiffer | Hematological | NHL | DLBCL | 4 | 0.086 | 0.021 | 0.189 | 0.036 |
| WSU-DLCL2 | Hematological | NHL | DLBCL | 3 | 0.093 | 0.009 | 0.095 | 0.009 |
| MOLM-13 | Hematological | Leukemia | M-AML | 3 | 0.105 | 0.012 | 0.136 | 0.013 |
| SU-DHL-16 | Hematological | NHL | DLBCL | 3 | 0.106 | 0.011 | 0.109 | 0.011 |
| HT | Hematological | NHL | DLBCL | 3 | 0.112 | 0.022 | 0.170 | 0.028 |
| U-2940 | Hematological | NHL | DLBCL | 5 | 0.113 | 0.032 | 0.178 | 0.045 |
| SU-DHL-4 | Hematological | NHL | DLBCL | 3 | 0.114 | 0.026 | 0.116 | 0.028 |
| DOHH-2 | Hematological | NHL | FL | 3 | 0.134 | 0.009 | 0.157 | 0.017 |
| OCI-LY-10 | Hematological | NHL | DLBCL | 3 | 0.141 | 0.021 | 0.188 | 0.023 |
| DB | Hematological | NHL | DLBCL | 3 | 0.151 | 0.058 | 0.173 | 0.054 |
| WSU-FSCCL | Hematological | NHL | FL | 3 | 0.156 | 0.020 | 0.190 | 0.024 |
| SU-DHL-6 | Hematological | NHL | DLBCL | 3 | 0.161 | 0.023 | 0.203 | 0.036 |
| SC-1 | Hematological | NHL | FL | 3 | 0.178 | 0.010 | 0.205 | 0.007 |
| OCI-LY-7 | Hematological | NHL | DLBCL | 3 | 0.186 | 0.020 | 0.246 | 0.017 |
| SU-DHL-10 | Hematological | NHL | DLBCL | 3 | 0.198 | 0.014 | 0.201 | 0.015 |
| REC-1 | Hematological | NHL | MCL | 3 | 0.199 | 0.023 | 0.260 | 0.029 |
| OCI-LY-3 | Hematological | NHL | DLBCL | 3 | 0.214 | 0.033 | 0.287 | 0.042 |
| OCI-LY-19 | Hematological | NHL | DLBCL | 3 | 0.224 | 0.036 | 0.355 | 0.045 |
| THP-1 | Hematological | Leukemia | M-AML | 3 | 0.227 | 0.066 | 0.569 | 0.095 |
| SU-DHL-5 | Hematological | NHL | DLBCL | 3 | 0.234 | 0.077 | 0.256 | 0.082 |
| HL-60 | Hematological | Leukemia | M-AML | 3 | 0.237 | 0.030 | 0.357 | 0.060 |
| JVM-2 | Hematological | NHL | MCL | 4 | 0.247 | 0.032 | 0.415 | 0.072 |
| Farage | Hematological | NHL | DLBCL | 3 | 0.265 | 0.020 | 0.278 | 0.025 |
| U-2932 | Hematological | NHL | DLBCL | 3 | 0.295 | 0.017 | 0.454 | 0.004 |
| KARPAS-299 | Hematological | ALCL | T-cell | 3 | 0.309 | 0.063 | 0.463 | 0.122 |
| SU-DHL-1 | Hematological | ALCL | T-cell | 3 | 0.323 | 0.013 | 0.393 | 0.017 |
| Mino | Hematological | NHL | MCL | 3 | 0.367 | 0.074 | 0.482 | 0.060 |
| Granta-519 | Hematological | NHL | MCL | 3 | 0.376 | 0.045 | 0.957 | 0.059 |
| JVM-13 | Hematological | NHL | MCL | 3 | 1.772 | 0.931 | 3.487 | 1.430 |
| HCC2157 | Solid | Breast | Basal | 3 | 0.052 | 0.014 | 0.091 | 0.025 |
| CAL-51 | Solid | Breast | Basal | 3 | 0.071 | 0.003 | 0.084 | 0.003 |
| MDA-MB-468 | Solid | Breast | Basal | 3 | 0.087 | 0.010 | 0.105 | 0.013 |
| CAL-85-1 | Solid | Breast | Basal | 3 | 0.089 | 0.015 | 0.169 | 0.006 |
| HCC70 | Solid | Breast | Basal | 3 | 0.094 | 0.005 | 0.183 | 0.006 |
| BT-20 | Solid | Breast | Basal | 3 | 0.116 | 0.003 | 0.225 | 0.021 |
| BT-549 | Solid | Breast | Basal | 3 | 0.146 | 0.031 | 0.219 | 0.049 |
| MT-3 | Solid | Breast | Basal | 3 | 0.150 | 0.041 | 0.467 | 0.088 |
| HCC1937 | Solid | Breast | Basal | 4 | 0.161 | 0.032 | 1.345 | 0.207 |
| HDQ-P1 | Solid | Breast | Basal | 3 | 0.162 | 0.030 | 0.375 | 0.105 |
| CAL-120 | Solid | Breast | Basal | 3 | 0.182 | 0.011 | 0.199 | 0.017 |
| MDA-MB-231 | Solid | Breast | Basal | 3 | 0.185 | 0.007 | 0.251 | 0.019 |
| HCC1806 | Solid | Breast | Basal | 3 | 0.187 | 0.020 | 0.386 | 0.057 |
| CAL-148 | Solid | Breast | Luminal | 3 | 0.187 | 0.031 | 0.298 | 0.038 |
| HCC1187 | Solid | Breast | Basal | 3 | 0.195 | 0.049 | 0.337 | 0.040 |
| DU4475 | Solid | Breast | Basal | 3 | 0.237 | 0.031 | 0.400 | 0.031 |
| MDA-MB-436 | Solid | Breast | Basal | 4 | 0.279 | 0.007 | 0.464 | 0.028 |
| HS578T | Solid | Breast | Basal | 3 | 0.286 | 0.014 | 0.331 | 0.018 |
| MB157 | Solid | Breast | Basal | 4 | 0.346 | 0.026 | 0.609 | 0.012 |
| HCC1143 | Solid | Breast | Basal | 3 | 0.400 | 0.074 | 0.606 | 0.089 |
| HCC38 | Solid | Breast | Basal | 4 | 0.430 | 0.048 | 0.524 | 0.067 |
| MDA-MB-157 | Solid | Breast | Basal | 3 | 0.472 | 0.021 | 0.714 | 0.085 |
| SNU-182 | Solid | Liver | Liver | 3 | 0.037 | 0.002 | 0.126 | 0.008 |
| HuH-7 | Solid | Liver | Liver | 3 | 0.053 | 0.012 | 0.077 | 0.019 |
| SNU-398 | Solid | Liver | Liver | 3 | 0.067 | 0.006 | 0.077 | 0.006 |
| SK-HEP-1 | Solid | Liver | Liver | 3 | 0.113 | 0.022 | 0.124 | 0.017 |
| Hep3B | Solid | Liver | Liver | 3 | 0.136 | 0.019 | 0.188 | 0.026 |
| HepG2 | Solid | Liver | Liver | 3 | 0.141 | 0.004 | 0.200 | 0.011 |
| PLC-PRF-5 | Solid | Liver | Liver | 3 | 0.144 | 0.009 | 0.205 | 0.009 |
| SNU-475 | Solid | Liver | Liver | 3 | 0.156 | 0.020 | 0.237 | 0.020 |
| SNU-423 | Solid | Liver | Liver | 3 | 0.163 | 0.027 | 0.251 | 0.031 |
| SNU-449 | Solid | Liver | Liver | 3 | 0.188 | 0.014 | 0.230 | 0.020 |
| SNU-387 | Solid | Liver | Liver | 3 | 0.309 | 0.047 | 0.406 | 0.050 |
| BHY | Solid | H&N |  | 2 | 0.040 | 0.010 | 0.210 | 0.052 |
| CAL-33 | Solid | H&N | Tongue | 3 | 0.043 | 0.002 | 0.070 | 0.005 |
| SCC-15 | Solid | H&N | Tongue | 3 | 0.057 | 0.009 | 0.679 | 0.121 |
| HN | Solid | H&N | Mouth | 3 | 0.117 | 0.009 | 0.178 | 0.011 |
| SCC-25 | Solid | H&N | Tongue | 3 | 0.127 | 0.024 | 0.179 | 0.025 |
| SCC-9 | Solid | H&N | Tongue | 3 | 0.129 | 0.035 | 0.363 | 0.054 |
| CAL-27 | Solid | H&N | Tongue | 3 | 0.144 | 0.021 | 0.177 | 0.033 |
| Detroit562 | Solid | H&N | Pharynx | 3 | 0.169 | 0.029 | 0.254 | 0.028 |
| RPMI-2650 | Solid | H&N | Nasal Septum | 3 | 0.219 | 0.036 | 0.389 | 0.049 |
| A253 | Solid | H&N | Salivary Gland | 3 | 0.303 | 0.032 | 0.378 | 0.053 |
| FADU | Solid | H&N | Pharynx | 3 | 0.793 | 0.052 | 1.162 | 0.013 |
| SK-LU-1 | Solid | Lung | NSCLC | 2 | 0.058 | 0.012 | 0.102 | 0.023 |
| NCI-H1395 | Solid | Lung | NSCLC | 5 | 0.061 | 0.013 | 8.070 | 1.930 |
| NCI-H2122 | Solid | Lung | NSCLC | 3 | 0.066 | 0.020 | 0.118 | 0.033 |
| NCI-H28 | Solid | Lung | NSCLC | 2 | 0.081 | 0.038 | 0.290 | 0.057 |
| NCI-H1993 | Solid | Lung | NSCLC | 2 | 0.085 | 0.034 | 0.222 | 0.068 |
| NCI-H1755 | Solid | Lung | NSCLC | 3 | 0.089 | 0.005 | 0.243 | 0.069 |
| NCI-H1944 | Solid | Lung | NSCLC | 2 | 0.091 | 0.031 | 0.299 | 0.077 |
| NCI-H1568 | Solid | Lung | NSCLC | 3 | 0.096 | 0.016 | 0.211 | 0.060 |
| NCI-H460 | Solid | Lung | NSCLC | 3 | 0.102 | 0.021 | 0.123 | 0.026 |
| NCI-H2030 | Solid | Lung | NSCLC | 3 | 0.105 | 0.011 | 0.162 | 0.015 |
| NCI-H596 | Solid | Lung | NSCLC | 3 | 0.122 | 0.014 | 0.364 | 0.031 |
| A549 | Solid | Lung | NSCLC | 65 | 0.130 | 0.005 | 0.161 | 0.007 |
| NCI-H2110 | Solid | Lung | NSCLC | 3 | 0.147 | 0.013 | 0.485 | 0.041 |
| Calu-3 | Solid | Lung | NSCLC | 7 | 0.151 | 0.028 | 0.565 | 0.202 |
| NCI-H2009 | Solid | Lung | NSCLC | 2 | 0.155 | 0.031 | 1.180 | 0.326 |
| NCI-H1703 | Solid | Lung | NSCLC | 3 | 0.157 | 0.055 | 0.457 | 0.107 |
| Calu-1 | Solid | Lung | NSCLC | 3 | 0.164 | 0.012 | 0.313 | 0.046 |
| NCI-H2228 | Solid | Lung | NSCLC | 2 | 0.164 | 0.041 | 0.454 | 0.104 |
| NCI-H226 | Solid | Lung | NSCLC | 3 | 0.170 | 0.030 | 0.385 | 0.039 |
| NCI-H647 | Solid | Lung | NSCLC | 3 | 0.180 | 0.024 | 0.513 | 0.030 |
| NCI-H1975 | Solid | Lung | NSCLC | 2 | 0.182 | 0.006 | 0.234 | 0.005 |
| NCI-H1299 | Solid | Lung | NSCLC | 3 | 0.183 | 0.013 | 0.239 | 0.013 |
| NCI-H727 | Solid | Lung | NSCLC | 3 | 0.183 | 0.064 | 0.921 | 0.428 |
| NCI-H1792 | Solid | Lung | NSCLC | 2 | 0.184 | 0.067 | 0.240 | 0.092 |
| NCI-H520 | Solid | Lung | NSCLC | 3 | 0.189 | 0.054 | 0.272 | 0.078 |
| EKVX | Solid | Lung | NSCLC | 3 | 0.208 | 0.048 | 0.334 | 0.056 |
| NCI-H2444 | Solid | Lung | NSCLC | 3 | 0.234 | 0.110 | 0.630 | 0.200 |
| Calu-6 | Solid | Lung | NSCLC | 3 | 0.245 | 0.039 | 0.927 | 0.285 |
| NCI-H838 | Solid | Lung | NSCLC | 2 | 0.249 | 0.023 | 0.354 | 0.013 |
| SW1573 | Solid | Lung | NSCLC | 2 | 0.252 | 0.008 | 1.615 | 0.766 |
| NCI-H1437 | Solid | Lung | NSCLC | 3 | 0.283 | 0.074 | 0.539 | 0.075 |
| NCI-H23 | Solid | Lung | NSCLC | 3 | 0.305 | 0.022 | 0.433 | 0.043 |
| HOP62 | Solid | Lung | NSCLC | 3 | 0.341 | 0.044 | 0.538 | 0.020 |
| NCI-H1563 | Solid | Lung | NSCLC | 3 | 0.342 | 0.218 | 1.812 | 1.272 |
| HOP92 | Solid | Lung | NSCLC | 8 | 0.356 | 0.102 | 0.852 | 0.191 |
| NCI-H358 | Solid | Lung | NSCLC | 3 | 0.417 | 0.035 | 0.688 | 0.024 |
| NCI-H1838 | Solid | Lung | NSCLC | 2 | 0.439 | 0.116 | 1.465 | 0.466 |
| NCI-H1650 | Solid | Lung | NSCLC | 2 | 0.714 | 0.033 | 1.073 | 0.196 |
| NCI-H441 | Solid | Lung | NSCLC | 3 | 1.043 | 0.332 | 3.721 | 1.564 |
